# A Literature Review of Interleukins in the Development and Treatment of Breast Cancer

**DOI:** 10.3390/ijms27083455

**Published:** 2026-04-12

**Authors:** Wiktoria Kraśnicka, Natasza Rybak, Kalina Wójcik, Aniela Stasiak, Maja Białowąs, Kamila Grzegorczyk, Tomasz Kolenda, Julian Malicki, Andrzej Marszałek, Marlena Janiczek-Polewska

**Affiliations:** 1 Faculty of Medicine, Poznan University of Medical Sciences, Fredry Street 10, 61-701 Poznan, Poland; 2 Research and Implementation Unit, Greater Poland Cancer Center, Garbary Street 15, 61-866 Poznan, Poland; 3 Microbiology Laboratory, Greater Poland Cancer Center, Garbary Street 15, 61-866 Poznan, Poland; 4 Department of Electroradiology, Poznan University of Medical Sciences, Garbary Street 15, 61-866 Poznan, Poland; 5 Department of Clinical Pathology, Poznan University of Medical Sciences and Greater Poland Cancer Center, Garbary Street 15, 61-866 Poznan, Poland; 6 Department of Clinical Oncology, Greater Poland Cancer Center, Garbary Street 15, 61-866 Poznan, Poland

**Keywords:** breast cancer, interleukins, cytokines, inflammatory process, carcinogenesis

## Abstract

Breast cancer is the most common malignant tumor in women worldwide. Triple-negative cancers have the worst prognosis, due to the low effectiveness of current therapies. In recent years, research has been conducted on the relationship between inflammatory process and the development of malignant tumors, including breast cancer. One of the elements influencing the inflammatory process is interleukins. These are small protein molecules belonging to the cytokine family that participate in the function of the human immune and hematopoietic systems. Interleukins are still being studied, and this is an area with significant knowledge gaps. More than 60 cytokines have been designated as interleukins over time, but not all of these designations are consistently used or universally accepted. In the available literature, we have only found information on 41. This is the first review to detail all 41 interleukins and their effects on breast cancer development. The review shows that interleukins affect the development of both locally advanced breast cancer and the development of distant metastases, mainly to the bones. Clinical trials are also underway in these areas: some have failed, and others are still ongoing. Due to the lack of success in the use of interleukins in the treatment of breast cancer, the latest strategies are based on combining several elements of the inflammatory process pathway occurring in breast cancer. This can probably bring us closer to therapeutic success in this area.

## 1. Introduction

Breast cancer is the most common cause of mortality among women diagnosed with cancer, but the number of deaths has declined in recent years, especially in Western populations, due to better screening techniques. Despite this, it remains the most commonly diagnosed cancer worldwide and the leading cancer in women (accounting for 24.5% of all cancers in women) [1,2]. In 2024, the estimated number of new breast cancer diagnoses in women was 310,720, which equates to 15.5% of all new cancer cases, while the number of breast cancer deaths was 42,250, representing 6.9% of all cancer deaths. Data collected from 2018 to 2021 indicated that 13.1% of women will be diagnosed with breast cancer during their lifetime (excluding 2020 due to the COVID-19 pandemic) [3]. Breast cancer incidence rates vary geographically and among different ethnic groups. Caucasian, white, non-Hispanic White individuals have been shown to have the highest incidence and mortality rates [4]. Cancer cells often go undetected by the immune system because they closely resemble the cells of the organism from which they originated, at the DNA and RNA levels. Cancer cells often evade detection by the immune system because they closely resemble the cells of the organism from which they originated, at the DNA and RNA levels. However, these cells are genetically and epigenetically altered. Furthermore, they evade the immune response through various mechanisms. Transformed cells can evade elimination through mechanisms such as immunoediting, downregulation of antigen expression (e.g., MHC class I), induction of immune tolerance, and establishment of an immunosuppressive tumor microenvironment. Among breast cancers, the most common group is those that originate in the cells lining the milk ducts, which are called ductal carcinomas, while others may start in the cells lining the lobules, in which case they are called lobular carcinomas. There are also a small number of cancers that originate in other tissues [5]. We can categorize breast cancers into two large groups: invasive/infiltrating cancers, in which the lesion has spread to the tissue surrounding the breast, and non-invasive/in situ cancers, where the lesion has not spread beyond the breast tissue where it began. The latter are often referred to as pre-cancerous [6].

Ductal non-invasive carcinoma (DCIS) accounts for 16% of all breast cancer diagnoses. Although it is not life-threatening, it increases the risk of developing invasive ductal breast cancer (IDC) later in life, and is thus considered a precursor to IDC, which is the most common type of breast cancer, accounting for about 80% of all invasive breast cancer cases. IDC starts in the milk ducts and then penetrates the wall of this duct, attacking the fatty tissue of the breast and other areas in the body.

Non-invasive lobular carcinoma (LCIS) is a benign breast condition characterized by an increase in the number of cells in the breast lobules. It has not spread beyond the lobules from which it originated, and despite its name, it is not considered true breast cancer. In contrast, invasive lobular breast cancer (ILC) is the second-most common type of breast cancer, accounting for approximately 10% to 15% of all invasive breast cancers [5,6]. Molecular classification of breast cancer, primarily based on gene expression profiling, categorizes tumors into four intrinsic subtypes—Luminal A (ER+ and/or PR+, HER2−, low Ki-67 (low proliferation)), Luminal B (ER+ and/or PR+, HER2− or HER2+, high Ki-67 (high proliferation)), HER2-enriched (ER- and PR-negative, HER2-positive (overexpression)), and Basal-like (triple-negative) (ER-, PR-, and HER2-negative). There are differences in prognosis, gene expression, and treatment response. These subtypes guide targeted therapies, such as hormone therapy for Luminal types, anti-HER2 agents for HER2-positive, and chemotherapy for TNBC. TNBC often has high-grade features. They are more common in younger women and BRCA1 mutation carriers, and tend to have a poorer prognosis [7].

Breast cancer can develop in a chronic inflammatory environment and can also trigger inflammatory responses in its microenvironment. One specific type of breast cancer is inflammatory breast cancer (IBC). This rare (1–5% of cases) but highly aggressive form of cancer is characterized by a rapid progression and the absence of a palpable tumor. In the context of cancer biology, the inflammatory process is often sterile and driven by damage-associated molecular patterns (DAMPs), genomic instability, oncogenic stress, and tumor-derived cytokines. Interactions between immune and non-immune cells are mediated by a group of cytokines called interleukins (ILs), which are signaling proteins. Their primary function is to control immune cells during immune and inflammatory responses, making them essential for the activation, differentiation, and migration of these cells [8]. Interleukins are also involved in the inflammatory process during tumorigenesis, influencing tumor progression through various mechanisms, particularly within the tumor microenvironment (TME) [9]. They can regulate their own renewal, additionally promoting resistance to chemotherapy by affecting the survival of cancer cells [10]. “Renewal” means that some interleukins (e.g., IL-7) can be bound to an internal receptor (internalized) by the cell and then recycled back to the cell surface, allowing for reuse. Additionally, some interleukins (e.g., IL-15, IL-24) induce the proliferation of NK cells and T lymphocytes, which, in a broader sense, means renewing the immune cell pool. Studies show that interleukins are associated with the development of breast cancer and contribute to disease progression through direct effects on tumor cells as well as the TME [11]. More than 60 cytokines have been designated as interleukins over time, but not all of these designations are consistently used or universally accepted [12,13,14]. Some candidates were later reclassified or not ‘formally recognized’ and widely discussed; many sources still mainly cover IL 1-38 (or IL-41, depending on when they were written and which classification they adopt). Here, we review recent developments on IL-1 to IL-41 in breast cancer, as most of the literature currently available concerns these proteins.

## 2. Interleukin 1 (IL-1)

IL-1 is a group of 11 pro-inflammatory cytokines. IL-1 is a glycoprotein with a molecular weight of 17 kDa. The main representatives of the IL-1 family are IL-1α, IL-1β, and the -1 receptor antagonist (IL-1RA) [15,16]. The ligands and receptors belonging to the IL-1 family are associated with both acute and chronic inflammation [17]. IL-1α and IL-1β are mainly produced by macrophages and monocytes, but can also be produced by endothelial cells, epithelial cells, fibroblasts, or cancer cells [10,18]. IL-1α and IL-1β bind and activate the widely prevalent type I IL-1 receptor (IL-1R1), triggering downstream signaling. IL-1RA also binds the IL-1RI receptor; it does not trigger signaling [18].

IL-1 cytokines and their receptors have been identified in the breast cancer tumor microenvironment [19]. IL-1α and IL-1β primarily expert pro-tumorigenic effects, although they may also induce anti-tumor immune response [20]. IL-1β is positively correlated with increased expression of other pro-inflammatory cytokines, including IL-8 or IL-6 [21,22]. Elevated IL-1 levels in the tumor microenvironment are associated with tumor progression, poorer patient prognosis, and increased resistance to cancer treatment [23,24]. IL-1 promotes the expression of metastasis-related genes, such as matrix metalloproteinases (e.g., *MMP-2*, *MMP-9*), in part by stimulating adjacent stromal and endothelial cells to produce growth factors and angiogenic mediators. This activity facilitates angiogenesis and metastasis [16]. Certain IL-1 and IL-1β variants have also been linked to breast tumorigenesis.

Guo et al. demonstrated that elevated IL-1β levels enhance breast tumor growth and lung metastasis, suggesting that targeting the IL-1 pathway may increase the efficacy of cancer treatment. The studies were conducted in animal and human breast cancer models [25]. It was found that IL-1β enhances the aggressiveness of luminal-type breast cancer cells (MCF7 cells) by increasing IL-6 production via a transglutaminase 2/NF-κB pathway. Anti-IL-1β or anti-IL-6 antibody treatment diminished epithelial-to-mesenchymal transition and stem-cell-like phenotypes in breast cancer cells [22]. In the MMTV-iFGFR1 transgenic mouse model, Reed et al. reported that IL-1β stimulates COX-2 expression, which contributes to early-stage mammary lesions [26]. IL-1β also enhances invasiveness in triple-negative breast cancer (TNBC). In vitro suppression of IL-1β expression using celastrol or zerumbone reduced TNBS cell proliferation [27,28]. In metastatic HER2-negative breast cancer, transcriptional signature of inflammation in blood leukocytes was attenuated following IL-1β blockade with the IL-1 receptor antagonist anakinra, which inhibits IL-1α and IL-1β binding to IL-1R. This study utilized a humanized mouse model [29]. In contrast, Castaño et al. reported in mice that high primary tumor IL-1β expression in lymph-node-positive breast cancer increased overall survival and reduced the incidence the of distant metastasis [30].

Precursor IL-1α in the tumor microenvironment increases tumor invasiveness and angiogenesis, thereby supporting tumor growth [31]. Kuan et al. reported that tumor-derived IL-1α, produced by breast cancer cells, acts on tumor-infiltrating myeloid cells, inducing the expression of thymic stromal lymphopoietin (TSLP) in these cells. TSLP functions as a critical survival factor for breast tumor cells by upregulating Bcl-2, and is required for lung metastasis. These findings were demonstrated in both in vitro and in vivo studies [32]. Liu et al. found in mice, that HER2 expression in the tumors induces the production of IL-1α- and IL-6-activating NF-κB and STAT3, which facilitates the generation and survival of cancer stem-like cells (CSCs). Pharmacological blockade of IL-1α signaling reduced the number of CSCs in tumors and improved chemotherapeutic efficacy [33]. In vitro studies conducted by You et al. indicated that IL-1α levels are also significantly increased in the triple-negative breast cancer [27]. Membrane-boundIL-1α expressed by certain cancer cells may enhance tumor cell immunogenicity, promoting the anti-tumor immune surveillance and tumor regression. Dagenais et al. found that IL-1R signaling may exert anti-tumor effect suppressing mammary tumor cell proliferation in the MMTV-PyMT breast cancer mouse model, which represents a luminal breast cancer. These findings suggest that IL-1α-IL1R signaling may have anti-tumor function in PyMT-driven breast cancer [34].

**Figure 1:**
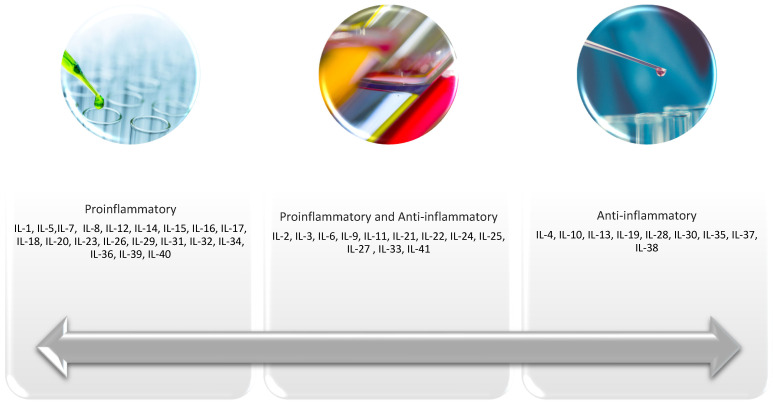
Interleukins can be classified according to their effects on the biological inflammatory response. Interleukins such as IL-1, IL-5, IL-7, IL-8, IL-12, IL-14, IL-15, IL-16, IL-17, IL-18, IL-20, IL-23, IL-26, IL-29, IL-31, IL-32, IL-34, IL-36, IL-39, and IL-40 exhibit pro-inflammatory effects. These pro-inflammatory interleukins initiate or intensify the inflammatory process, promote the immune response, recruit immune cells to sites of infection or injury, and stimulate the production of acute-phase proteins. In contrast, interleukins such as IL-4, IL-10, IL-13, IL-19, IL-28, IL-30, IL-35, IL-37, and IL-38 demonstrate anti-inflammatory effects by inhibiting inflammation and limiting excessive immune responses, thereby preventing tissue damage. Additionally, interleukins including IL-2, IL-3, IL-6, IL-9, IL-11, IL-21, IL-22, IL-24, IL-25, IL-27, IL-33, and IL-41 possess both pro-inflammatory and anti-inflammatory properties.

**Figure 2:**
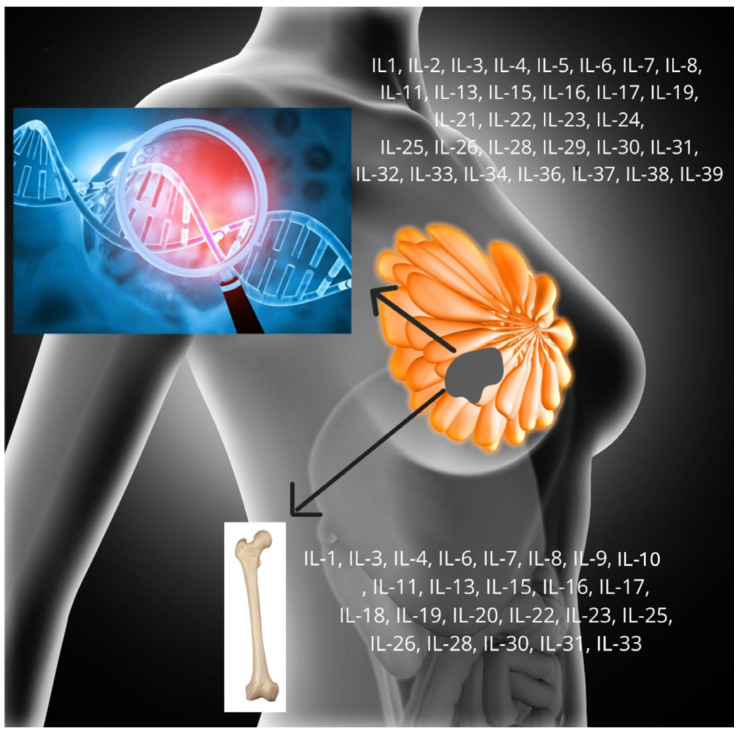
A range of interleukins, including IL-1, IL-2, IL-3, IL-4, IL-5, IL-6, IL-7, IL-8, IL-11, IL-13, IL-15, IL-16, IL-17, IL-19, IL-21, IL-22, IL-23, IL-24, IL-25, IL-26, IL-28, IL-29, IL-30, IL-31, IL-32, IL-33, IL-34, IL-36, IL-37, IL-38, and IL-39, influence local and locoregional growth in breast cancer. Additionally, IL-1, IL-3, IL-4, IL-6, IL-7, IL-8, IL-9, IL-10, IL-11, IL-13, IL-15, IL-16, IL-17, IL-18, IL-19, IL-20, IL-22, IL-23, IL-25, IL-26, IL-28, IL-30, IL-31, and IL-33 are implicated in the development of distant metastases, particularly bone metastases, in breast cancer. IL refers to interleukin.

**Figure 3:**
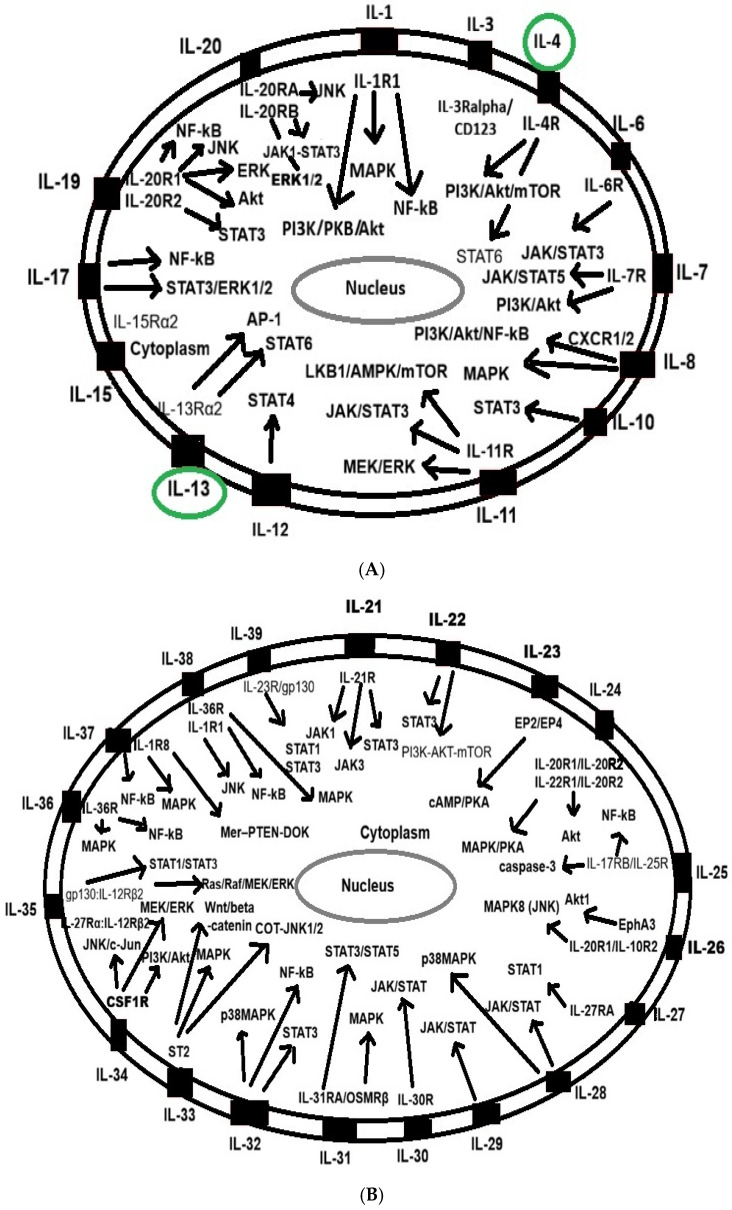
(**A**) Known and potential interleukin signaling pathways in breast cancer. Green indicates that interleukin IL-4 and IL-13 are structurally and functionally related. (**B**) Known and potential interleukin signaling pathways in breast cancer.

Targeting IL-1 signaling with agents such as anakinra, antibody treatment against IL-1β (canakinumab), suppression of IL-1β expression (using celastrol or zerumbone) or administration of inflammasome inhibitors may enhance the efficacy of breast cancer treatment [20,22,27,28]. Zhou et al. reported that anakinra or kanakinumab prevented bone metastases in breast cancer in both in vitro and in vivo models; however, in some cases, primary breast tumor growth was also observed. Additionally, targeting IL-1 signaling with MLX01 (IL-1β secretion inhibitor) reduced both primary tumor growth and bone metastasis, suggesting a potential new therapeutic strategy for breast cancer bone metastasis [35]. In contrast, Mattarollo et al. found that in experimental breast adenocarcinomas and fibrosarcomas induced de novo by carcinogen in mice, IL-1β inhibitors diminished the therapeutic effect of anthracyclines [36] (Figure 1, Figure 2 and Figure 3A) (Table 1).

## 3. Interleukin 2 (IL-2)

IL-2 is a small cytokine with a molecular weight of 15 kDa and exerts pleiotropic effects on the immune system. IL-2 receptor (IL-2R) is a heterotrimeric protein expressed on the surface of lymphocytes that binds to and responds to IL-2. IL-2R comprises three subunits: IL-2Rα (CD25), IL-2Rβ (CD122), and the common γc (CD132). These receptor chains are expressed separately and differentially across cell types, assembling in various combinations and orders to form low-, medium-, and high-affinity IL-2 [37,38]. Various modifications and designs of IL-2, such as PEG modification, Fc fusion, and other strategies, have been developed by biotechnology companies to optimize IL-2 efficacy [37,38,39]. IL-2 is a pro-inflammatory cytokine that activates T helper cells, cytotoxic T cells, B cells, natural killer (NK) cells, and macrophages [40]. It is recognized as one of the first effective immunotherapies for solid tumors. Human recombinant IL-2 (Proleukin^®^, Novartis, Basel, Switzerland) received approval from the U.S. Food and Drug Administration (FDA) for the treatment of metastatic renal cell carcinoma in 1992 and metastatic melanoma in 1998. However, IL-2 therapy is limited by a broad toxicity profile, including alterations in mental status, pulmonary congestion, renal dysfunction with oliguria or anesthesia, and hypotension resulting from capillary leak syndrome [41]. Low IL-2 levels are associated with increased breast cancer recurrence. Furthermore, IL-2influences the development and initiation of breast cancer. Muraro et al. demonstrated significantly lower IL-2 levels in patients with overexpression of epidermal growth factor receptor 2 (HER-2) and locally advanced breast cancer in an in vitro study. Polymorphisms in the *IL-2* gene are linked to an increased incidence of breast cancer and may serve as prognostic markers [42]. Conversely, Garcia-Tuñón et al. reported higher expression of IL-2 and its receptors in infiltrating breast tumors compared to in situ surgical specimens [37] (Figure 1 and Figure 2) (Table 1).

## 4. Interleukin 3 (IL-3)

IL-3 (also called MCGF, MGC79398, MGC79399, MULTI-CSF) is encoded by a gene on chromosome 5 at position 5q31.1, located only 9 kilobases from the *GM-CSF* gene, and shares functional similarities with GM-CSF. The human *IL-3* gene encodes a 152 amino acid protein [43,44,45]. It is secreted by T lymphocytes. IL-3 influences hematopoiesis and enhances the immune system’s response to disease. As a multipotent hematopoietic growth factor, IL-3 supports the proliferation and differentiation of early hematopoietic progenitors, particularly within myeloid and some lymphoid lineages. However, IL-3 acts similarly to interleukin 5, as both cytokines promote their proliferation, survival, and activation of basophils and eosinophils. The similarity in their actions reflects the fact that their receptors share a common protein subunit (βc), which activates similar intracellular signaling pathways. The IL-3 receptor is a heterodimer composed of an IL-3-specific α-chain and the common β-chain [45,46]. In healthy individuals, IL-3 does not participate in hematopoiesis and is secreted only during inflammatory processes, thereby stimulating blood cell production during infection. This distinguishes IL-3 from granulocyte–macrophage colony-stimulating factor [47]. Basal-like breast cancers, typically triple-negative and accounting for 10–15% of all breast cancers, are largely unresponsive to targeted therapy, resulting in high rates of metastasis and mortality. In this subgroup, elevated expression of IL-3 and IL-3 receptor subunits (IL-3RA + CSF2RB) has been demonstrated. IL-3 expression correlates with poor treatment outcomes, although receptor expression does not show this association [48] (Figure 1, Figure 2 and Figure 3A) (Table 1).

## 5. Interleukin 4 (IL-4)

IL-4 (also known as IL4, BCGF1, BSF1) is a cytokine that induces the differentiation of naive helper T cells (Th0) into Th2 cells. The interleukin-4 receptor also binds IL13, resulting in many shared functions between these interleukins [49]. STAT6 is a key mediator of the immune regulatory signals of IL-4 [50]. Additionally, IL-4 is essential for the development of innate CD8 + T cells in the thymus of several mouse stains deficient in the genes such as *Itk*, *KLF2*, *CBP*, and *Id3*, even without prior antigen exposure [51]. The literature presents conflicting evidence regarding the role of IL-4 in breast cancer development. Some studies report anticancer effects of IL-4, while others suggest pro-cancer effects in breast cancer [51,52,53,54,55]. IL-4 can induce apoptosis and inhibit growth in cultured breast cancer cells, indicating a potential anticancer role [54,55]. It also regulates enzymes involved in estrogen synthesis, which is significant for breast cancer development [53]. Conversely, other studies demonstrate a pro-tumorigenic effect of IL-4, as it can promote cancer cell proliferation and interact with various cells in the tumor microenvironment, including lymphocytes, fibroblasts and endothelial cells, thereby facilitating tumor growth. IL-4 has also been shown to enhance tumor progression in TNBC cell lines by increasing glucose uptake and metabolism [55]. Furthermore, IL-4 contributes to cancer cell survival and migration, promoting breast cancer metastasis [52,55]. Notably, in mouse model, agents targeting IL-4R have been shown to reduce breast cancer metastasis [52] (Figure 1 and Figure 2) (Table 1).

## 6. Interleukin 5 (IL-5)

IL-5 a cytokine secreted by Th2 CD4^+^ lymphocytes and mast cells. IL-5 is best known for regulating the growth, differentiation, activation, and survival of eosinophils. It is the key cytokine driving eosinophil lineage commitment and peripheral expansion. Eosinophil development is more closely linked to IL-5 (and, to a lesser extent, IL-3 and GM-CSF). IL-5 promotes B lymphocyte proliferation and stimulates antibody production, mainly IgA [56,57]. Human IL-5 consists of 115 amino acids. IL-5, in its active form, is a homodimer, unlike IL-3 and G-CSF [57,58]. Drugs acting against IL-5 include benralizumab, mepolizumab and reslizumabv [58,59]. In ER- and TNBC-type breast cancer patients, elevated IL-5 levels may be associated with poorer survival [59]. IL 5 influences eosinophil activity, which in certain contexts, may contribute to tumor growth and metastasis. At the same time, IL 5 produced by CD4^+^ T cells and eosinophils can enhance the efficacy of immune checkpoint blockade (ICB) in breast cancer by promoting eosinophil expansion, increased IL 5 production, and subsequent CD8^+^ T cell activation. Thus, carefully modulating IL 5 or its receptor, rather than simply inhibiting it, may allow fine tuning of eosinophil responses and potentially improve outcomes in breast cancer [60] (Figure 1 and Figure 2) (Table 1).

## 7. Interleukin 6 (IL-6)

IL-6 is a cytokine released by various macrophages and monocytes during normal immune system function, mediating responses to injuries, autoimmune diseases, infections, and inflammatory conditions. It is also involved in processes such as cell differentiation, hematopoiesis, neuroprotection, tissue remodeling and cancer development [10,61]. IL-6 binds to its specific receptor (IL-6Rα), and this complex then associates with gp130, the shared signal-transducing subunit of the IL-6 cytokine family, initiating JAK/STAT signaling [62]. Binding to gp130 is only possible when IL-6 is complexed with IL-6R. Unlike gp130, which is ubiquitously produced in all cells, IL-6R expression has been detected in only a few locations, one of which is the mammary gland [63,64]. The complete IL-6 signaling complex is formed as a hexamer: [IL-6/IL-6R/gp130] × 2. This complex promotes epithelial-to-mesenchymal transition (EMT) and the acquisition of cancer stem-like properties [63,65]. Excessive IL 6 secretion activates the JAK2/STAT3 signaling pathway, leading to STAT3 phosphorylation and activation, an oncogenic transcription factor that promotes key cancer-associated features, including invasion and metastasis, immunosuppression, angiogenesis, and metabolic reprogramming [63,66]. The IL 6/JAK2/STAT3 axis therefore plays a central role in driving metastatic and invasive behavior in malignant tumors [67]. This pathway is also responsible for the strong suppression of the immune system’s anti-tumor response [68]. Additionally, STAT3 enhances IL-6 signaling, further promoting inflammation [67]. Chronic inflammation is estimated to contribute to the development of approximately 25% of cancers in adults [69].

IL-6 is also implicated cancer development by activating fibroblasts, facilitating communication between cancer cells and cancer-associated fibroblasts (CAFs). A strong correlation exists between the tumor microenvironment (TME), where CAFs are essential components, and the pathogenesis of various cancer types. CAFs promote tumor progression by suppressing the host’s cellular immune response, producing growth factors, and supporting angiogenesis [62]. Additionally, IL-6 is produced by several TME-associated cells, including adipocytes, lymphatic endothelial cells, and Myeloid-Derived Suppressor Cells (MDSCs) [70]. IL-6 contributes to immune evasion by increasing programmed death ligand 1 (PD-L1) levels, the primary ligand for programmed death 1 (PD-1). Under physiological conditions, the PD-1/PD-L1 interaction prevents excessive immune system activation and protects against autoimmune diseases. In cancer, however, this interaction facilitates tumor cell immune evasion [71,72]. Numerous studies have demonstrated IL-6 secretion by breast cancer cell lines, with secretion levels varying by breast cancer subtype. ER- cells secrete higher levels of IL-6 than ER+ cells, a difference associated with the estrogen receptor’s disruption of NF-κB transactivation, which inhibits IL-6 expression. This is seen in IL-6 receptor (IL-6R), while ER- cells primarily express the membrane-bound isoform. Despite low IL-6 secretion, ER+ cells are highly sensitive to IL-6 in the tumor microenvironment [63]. In summary, IL-6 plays a critical role in breast cancer development and progression, exerting pleiotropic effects depending on secretion context and varying among patients according to subtype (Figure 1, Figure 2 and Figure 3A) (Table 1).

## 8. Interleukin 7 (IL-7)

IL-7 was first identified in 1988 as a growth factor for B cell precursors in mouse bone marrow culture. Recombinant human IL-7 is a 17.4 kDa protein consisting of 153 amino acid residues. The *IL7* gene is located on chromosome 8q12-q13, and the protein is a glycoprotein with four a-helices and a hydrophobic core [73,74]. IL-7 is essential for lymphocyte survival and development, and it is involved in the proliferation and rearrangement of certain T cell receptor (TCR) genes in early thymic stem cells [73]. It is also critical for the survival and homeostatic proliferation of mature T lymphocytes after thymic egress [75]. IL-7 participates in the host response to HIV by stimulating the differentiation of cytotoxic lymphocytes, which promote apoptosis of virus-infected cells [76]. In patients undergoing allogeneic hematopoietic stem cell transplantation (HSCT), elevated plasma IL-7 levels in the early post-transplant period are associated with reduced T cell numbers and increased risk of acute graft-versus-host disease (aGVHD) and mortality. IL-7 has also been implicated in the pathogenesis of Graves’ disease [75,77]. In breast cancer, IL-7 has been associated with both promotion and inhibition of tumor growth and metastasis. The cytokine can induce breast cancer cell growth in vitro via a wortmannin-sensitive pathway, and can stimulate invasion and metastasis [78]. IL-7 was accompanied by IL-15 in stimulating breast cancer cell invasion. In vitro studies have shown that, the IL-7 splice variant IL-7δ5 has been shown to induce metastasis in breast cancer cells by activating the PI3K/Akt pathway [79]. Moreover, it induces epithelial-to-mesenchymal transition (EMT). Serum IL-7 levels are significantly higher in patients with early invasive breast cancer compared to healthy controls [80]. Conversely, IL-7 may also exert anti-tumor effects By supporting immune cel development and function within the tumor microenvironment. Both in vitro and in vivo studies indicate that IL-7 enhances the therapeutic effect of CAR-T cells, especially in triple-negative breast cancer, and can be combined with other anticancer drugs to improve efficacy [81]. IL-7 production from lymphatic endothelial cells is stimulated by TGFβ1, potentially contributing to metastasis [82] (Figure 1, Figure 2 and Figure 3A) (Table 1).

## 9. Interleukin 8 (IL-8)

IL-8, also known as CXCL8, is a chemokine involved in both that takes part in endocrine and paracrine tumor promotion. It attracts multinucleated leukocytes with inflammatory properties, making it a valuable tumor marker [83]. Tumors frequently produce IL-8, which supports angiogenesis, survival of cancer stem cells, local release of growth factors, and recruitment of bone marrow cells with immunosuppressive functions [10]. Overproduction of pro-inflammatory cytokines, such as IL-8, promotes inflammation that drives tumor growth. Elevated serum levels of IL-8, along with TNF-∝ and IL-6, are associated with tumor progression and poor prognosis, with a particularly strong association observed between IL-8 and TNF in cancer patients [84]. Oxidative stress also increases IL-8 production in cancer cells, further contributing to its role as an angiogenic factor [85]. In breast cancer, elevated IL-8 expression is observed in ER- and HER2+ subtypes, but it also enhances aggressiveness and metastatic potential in ER+ cancer cells [10,83]. An inverse correlation exists between ER receptor levels and IL-8, and although ER- cancer cells produce more IL-8, the underlying mechanism remains unclear. The ER receptor and cancer cell growth factor are influenced by the mitogen-activated protein kinase (MAPK cascade), which is a part of the IL-8 signaling pathway [85,86]. In studies of IL-8-induced TNBC cell invasion following proteasome inhibition by bortezomib (BZ), BZ increased TNBC cell invasiveness, which was reduced by IκB kinase (IKK) inhibition, leading to decreased IL-8 expression [87]. Evidence that IL-8, synthesized in cancer-associated adipocytes, significantly contributes to tumor immune suppression, growth and metastasis. It may also remodel the tumor microenvironment by inhibiting CD4+ and CD8+ T lymphocyte infiltration and increasing CD274 (PD-L1) expression in TNBC syngraft models [88] (Figure 1, Figure 2 and Figure 3A) (Table 1).

## 10. Interleukin 9 (IL-9)

IL-9, synonym Cytokine P40, is produced by activated Th2 lymphocytes. The gene encoding IL-9 is located on chromosome 5 [89,90]. IL-9 activates mast cells and increases their proliferation, and also acts on B lymphocytes, T lymphocytes, macrophages, eosinophils, hematopoietic precursor cells, epithelial cells and neurons. It is involved in responses to parasitic infections and the pathogenesis of asthma [89,90,91,92]. Additionally, it participates in oncogenesis. In breast cancer, IL-9, an interleukin, may play anti-tumor role. Physiological three-dimensional invasion assays have shown that IL-9 inhibits the metastatic potential of breast (MDA-MB-231 and MCF-7) and cervical (HeLa) cancer cells by regulating extracellular matrix remodeling and cellular contractility. Analyses of IL-9-dependent pathways in cancer cell metastasis, including proteolysis, contractility, and focal adhesion dynamics, have elucidated mechanisms underlying IL-9-mediated suppression of invasion. IL-9 blocks tumor cell-collagen degradation, highlighting its effects on extracellular matrix remodeling. Additionally, IL-9 suppresses IL-17- and IFN-γ-induced metastasis of both human breast and cervical cancer cells [93]. Another study investigated the effect of IL-9 on macrophage M1 polarization and confirmed its anti-tumor potential by retraining Tumor-Associated Macrophages (TAMs) and promoting chemokine secretion. IL-9 stimulates macrophage proliferation and polarizes them toward the pro-inflammatory M1 phenotype, a process dependent on IFNγ. Tumor-localized IL-9 also polarizes TAMs toward M1 in vivo. Furthermore, IL-9 induces TAMs to release CCL3/4 and CXCL9/10, recruiting anti-tumor immune cells, including T and natural killer cells, into the tumor microenvironment. Peritoneal treatment with recombinant IL-9 delayed the growth of macrophage-enriched 4T1 breast cancer in syngeneic mice, whereas IL-9 treatment did not reduce tumor growth in the absence of macrophage enrichment. These findings demonstrate the efficacy of IL-9 in macrophage polarization to trigger anti-tumor immunity [94] (Figure 1 and Figure 2) (Table 1).

## 11. Interleukin 10 (IL-10)

IL-10 is a cytokine located on chromosome 1 that plays an immunomodulatory and anti-inflammatory roles in the human immune system [95,96]. It is primarily expressed by T lymphocytes, natural killer (NK) lymphocytes and macrophages [97]. IL-10 binds to a receptor complex consisting of two chains, IL-10R1 and IL-10R2, both members of the class II receptor family [98,99]. Binding of IL-10 to IL-10R1 leads to phosphorylation of Janus Kinase-1 (JAK1) and Tyrosine Kinase-2 (TYK-2), resulting in inactivation of Signal Transducer and Activator of Transcription-3 (STAT3), which translocates to the nucleus and induces transcription of genes involved in cell cycle progression and anti-apoptotic [98]. IL-10 also inhibits the synthesis of other cytokines, including IL-1α, IL-1β, IL-6, IL-8, IL-12, and IL-18 [100]. The IL-10 promoter contains several single-nucleotide polymorphisms (SNPs), such as an adenine to guanine substitution at nucleotide-1082 (rs1800896), a thymine to cytosine substitution at nucleotide-819 (rs1800871), and an adenine to cytosine substitution at nucleotide-592 (rs1800872). Studies have investigated whether these SNPs are associated with breast cancer risk in specific populations [95,101]. Meta-analysis indicate that IL-10 rs1800896 and rs1800872 (AA vs. CC; A vs. C) polymorphisms significantly increase BC risk in Asians, while rs1800871 and rs1800872 (A vs. C) are associated with risk in Caucasians [95]. IL-10 is correlated with breast cancer and exhibits both pro- and anti-tumor properties [102]. It exerts diverse effects on tumor cells [103]. IL-10 stimulates CD8+ T lymphocytes, leading to increased IFN-γ production, which has cytotoxic effects on tumor cells [103]. Conversely, IL-10 inhibits antigen presentation by myeloid cells, suppressing certain cytokine production and potentially restraining IFN-γ levels [103]. IL-10 also inhibits production of other cytokines, such as IL-23, which promotes tumor growth; thus, IL-10-mediated downregulation of IL-23 can limit cancer progression [103]. The role of IL-10 in cancer development remains unclear [103]. Some studies suggest that IL-10 downregulates cancer progression in mice through anti-metastatic and anti-tumor properties [104]. Others describe a possible correlation between the levels of IL-10 in patients’ serum and the stage of the disease [105]. Additional reports indicate associations between IL-10, Bcl-2 and Bax [106]. IL-10 presented an inverse relationship with p53 [106]. Low expression of IL-10 in breast tumor and stromal cells has been linked to unfavorable prognosis [107]. Anti-tumor and anti-metastatic effects of IL-10 have also been observed in breast cancers [108]. Some cancer therapies target programmed cell death-1 (PD-1) and programmed cell death ligand 1 (PD-L1), which inhibit anti-tumor immunity [100,109]. Pembrolizumab (a PD-1 inhibitor) and atezolizumab (a PD-L1 inhibitor) are used in breast cancer immunotherapy [100,110,111]. According to Lamichhane et al., in patients resistant to anti-PD-1/anti-PD-L1 monotherapy, there is an association between IL-10, PD-1 and PD-L1 signaling pathways. Consequently, combined blockade of PD-1 and IL-10 reduces tumor burden and enhances immune system efficacy, contributing to improved prognosis in ovarian cancer patients [100,109] (Figure 1, Figure 2 and Figure 3A) (Table 1).

## 12. Interleukin 11 (IL-11)

IL-11 is a cytokine first isolated in 1990 from bone marrow stem cells, specifically from fibrocytes [112]. IL-11 activates megakaryocytes and B lymphocytes. Additionally, it acts on T lymphocytes by stimulating the production of Th2 cytokines and inhibiting the production of Th1 cytokines [112]. Additionally, IL-11 promotes osteoclast formation by activating the STAT3 pathway and, potentially, independently of RANKL [113]. Expression of IL-11 is often associated with poorer prognosis in breast cancer patients. Furthermore, high levels of IL-11 correlate with an increased risk of bone metastasis [114]. IL-11 is also associated with tumor progression and may stimulate tumor cell survival and proliferation, angiogenesis, and invasion. In an in vitro study, piR-2158-IL11 was shown to inhibit angiogenesis in breast cancer. piR-2158 is a transcriptional repressor of IL-11 by competing with the AP-1 transcription factor subunit FOSL1 for binding to the IL-11 promoter. STAT3 signaling mediated the regulation of cancer cell stemness and tumor growth by piR-2158-IL11. Interestingly, curcumin, may help modulate IL-11 signaling and potentially enhance the efficacy of chemotherapy [115]. Given its potential role in breast cancer progression and involvement in bone metastasis, IL-11 is being investigated as a potential therapeutic target [114] (Figure 1, Figure 2 and Figure 3A) (Table 1).

## 13. Interleukin 12 (IL-12)

IL-12 is a cytokine produced by phagocytes, dendritic cells and other antigen-presenting cells, including B lymphocytes. IL-12 is a heterodimer with a molecular weight of about 75 kDa. IL-12 initiates T lymphocyte differentiation towards Th1 cells and inhibits Th2 lymphocytes activity [116,117,118]. Additionally, it activates NK cells, macrophages and monocytes. IL-12, acting together with IFN-γ and IL-2, directs T helper cell responses towards the TH1 phenotype rather than the TH2 phenotype. In addition, IL-12 is also important in the immune response to viral diseases and has significant anti-tumor activity [116,118]. Derin et al. did not show any difference in IL-12 expression in breast cancer patients and healthy controls [119]. However, in contrast, genetic variation in the IL-12 pathway (e.g., single-nucleotide polymorphisms (SNPs) in *IL12A*, *IL12B*, *IL12RB1*, *IL12RB2*, *STAT4*, *JAK2*, *TYK2*) has been associated with altered breast cancer risk in several populations, including Puerto Rican women, suggesting that inherited differences in IL-12 signaling can modulate susceptibility [120]. Moreover, serum IL-12 has been investigated as a potential diagnostic or prognostic marker in breast cancer, with some studies linking lower IL-12 to more advanced disease or worse prognosis [121]. In addition, IL-12 has been explored as an immunotherapy in breast cancer and especially in triple-negative breast cancer (TNBC), including combinations with chemotherapy, anti-PD L1, or agents that deplete Myeloid-Derived Suppressor Cells, to enhance NK cell and CD8+ T cell responses in the tumor microenvironment [122] (Figure 1 and Figure 3A) (Table 1).

## 14. Interleukin 13 (IL-13)

IL-13 is a cytokine produced primarily by Th2 lymphocytes, with lesser contributions from NK cells and eosinophils. Human IL-13 is a 17 kDa glycoprotein encoded on chromosome 5. IL-13 exhibits pleiotropic anti-inflammatory and immunoregulatory effects [14]. It is implicated in a range of diseases, including asthma, parasitic infections, nephrotic syndrome, atopy, gastrointestinal diseases, arthritis, and several cancers such as breast, ovarian, pancreatic, colon, head and neck, bladder and Hodgkin’s lymphoma [14,123,124,125]. Evidence suggests that IL-13 may have both tumor-promoting and tumor-inhibiting roles [14]. In breast cancer, IL-13 demonstrates a dual function, influencing both tumor growth and metastasis. IL-13 expression is elevated in tumors compared to adjacent tissue or benign breast disease. Its receptor, IL-13Rα2, is overexpressed in metastatic breast cancer and is associated with poorer prognosis [126]. In vitro and in vivo studies indicate that high IL13Rα2 expression in brain metastases from breast cancer predicts reduced survival. IL13Rα2 promotes proliferation while suppressing invasion [127]. Expression of IL13Ralpha2 is significantly increased in basal breast cancers compared to luminal primary breast tumors and in certain metastatic basal–B breast cancer cell lines. Overexpression of IL13Ralpha2 correlates with poorer survival in nonmetastatic breast cancer patients. Depletion of IL13Ralpha2 in metastatic breast cancer cells slightly delays primary tumor growth but markedly inhibits lung metastasis in vivo. Silencing IL13Ralpha2 impairs the migratory capacity of metastatic breast cancer cells. Furthermore, IL-13 treatment and IL13Ralpha2 downregulation in a STAT6-dependent manner increases TP63 expression, a metastasis suppressor. These findings suggest that the STAT6-TP63 pathway may limit the metastatic spread of breast cancer cells to the lung [128] (Figure 1, Figure 2 and Figure 3A) (Table 1).

## 15. Interleukin 14 (IL-14)

IL-14, also known as taxilin, has been identified as a B-cell growth and proliferation factor. IL-14 is a high-molecular-weight (HMW-BCGF). IL-14 in humans is encoded by the *TXLNA* gene. It is produced by T cells and some malignant B lymphocytes [129]. The exact role of this cytokine is still uncertain. It is implicated in the pathogenesis of autoimmune diseases [129]. The role in the development of breast cancer is unknown at present (Figure 1) (Table 1).

## 16. Interleukin 15 (IL-15)

IL-15 is secreted mainly by dendritic cells, monocytes and macrophages. It is a glycoprotein with a molecular weight of 14–15 kDa. IL-15 is encoded by the *IL15* gene in humans. It is a cytokine with a wide range of effect. IL-15 affects the immune system, both innate and adaptive mechanisms [130,131]. IL-15 induces NK cell proliferation. In addition, it activates neutrophils, induces angiogenesis, acts on activated T lymphocytes, and participates in the maintenance of memory T lymphocytes and in the differentiates of dendritic cells. IL-15 is generally similar to IL-2, but it differs in its effects on the immune system. IL-15 is associated with the pathogenesis of various diseases, including cancers. In combination with specific antibody therapy, it can demonstrate anti-tumor activity [130,131,132]. IL-15 affects the immune system in breast cancer, especially NK (natural killer) cells and CD8 T cells. These cells are crucial for tumor immunosurveillance. First, it inhibits tumor growth and metastasis by acting on the immune system, mainly in NK cells [133,134]. On the other hand, it promotes metastasis [131,135]. To date, IL-15 is being investigated as a potential therapeutic target through the use of IL-15 agonists and their combination with other therapies to enhance anti-tumor immunity. Additionally, some studies have shown that monitoring IL-15 levels can help in assessing prognosis and response to treatment [136]. Preclinical studies in a mouse model show that IL-15 complexes significantly reduce tumor growth and metastasis, especially when combined with radiotherapy or monoclonal antibodies (like trastuzumab) [137,138] (Figure 1, Figure 2 and Figure 3A) (Table 1).

## 17. Interleukin 16 (IL-16)

IL-16 is a cytokine produced by various immune cells, including T cells, B cells, macrophages, eosinophils, and mast cells [139]. Its natural receptor is CD4. IL-16 is considered to have pro- and anti-tumor effects on breast cancer development. It was found that IL-16 enhances breast cancer progression by promoting macrophage accumulation in the sites of the tumor [140]. IL-16 is also considered a risk factor for HER2-positive breast cancer. However, Wen et al. showed that increased IL-16 expression correlates with inhibited tumor growth and a better prognosis in mice. When the tumors became palpable, mice were administered recombinant IL-16. Compared with control mice, IL-16 treated mice developed significantly smaller tumors. On the other hand, neutralization of IL-16 with an anti-IL-16 antibody (αIL-16) accelerated tumor growth. Moreover, they collected serum samples from patients diagnosed with breast cancer and found that serum IL-16 concentrations were significantly lower in cancer cohorts compared to healthy individuals. It suggests that tumor presence may inhibit local or systemic IL-16 production. Next, they performed single-cell RNA sequencing (scRNA-seq) on human breast cancer tissues and the corresponding normal tissues. High levels of IL-16 were detected in immune clusters, including T cell, B cell, monocyte, and macrophage clusters, whereas tumor and stromal cells expressed very low levels of IL-16 [139] (Figure 1 and Figure 2) (Table 1).

## 18. Interleukin 17 (IL-17)

IL-17 is synthesized primarily by Th17 cells, but also by CD8 Tc17 cells, natural killer cells, and lymphoid tissue inducer cells. It is a pro-inflammatory cytokine. The IL-17 family consists of six members, designated IL-17A-IL-17F. IL-17 cytokines have a pro-tumor effect by acting in one of two ways—either directly on tumor cells or indirectly by influencing the patient’s anti-tumor response and altering the microenvironment, which enhances the disease’s invasive and metastatic characteristics. These cytokines are involved in inflammatory, autoimmune diseases, or malignant neoplasms [10,16]. Each member of the IL-17 family transmits signals through a distinct group of receptors known as interleukin-17 receptors (IL-17R) [141]. IL-17A and IL-17F are the most closely related members of the IL-17 family, as they are co-expressed on linked genes and co-produced by Type 17 cells. Likewise, there are five receptor subunits: IL-17RA to IL-17RE. IL-17RA serves as the shared receptor subunit for most IL-17 family ligands, forming heterodimers with ligand-specific partners (e.g., IL-17RC for IL-17A/F). This cytokine enhances the expression of inflammatory genes by either initiating de novo gene transcription or stabilizing target mRNA transcripts [142]. Th17 cells play a key role in breast cancer through the inflammatory process, by producing cytokines such as IL-17, IL-22, IL-21, IL-9 (and TNFα in some contexts), which enhance breast cancer progression, growth, and proliferation [143]. Studies have shown that the Th17 population significantly increases in the peripheral blood of breast cancer patients compared to healthy individuals, contributing to cancer progression [144]. Chronic inflammation may lead to an increased risk of developing breast cancer. David N. Danforth demonstrated in his publication that inflammatory changes are observable in a healthy breast tissue with increased breast cancer risk, as well as in adipocytes and crown-like structures (CLS), which may be related to chronic inflammation [145]. But (CLS) in healthy breast tissue links obesity/inflammation to risk, but it is not IL-17-specific. CLS produce TNFα/IL-6/IL-1β primarily; IL-17 link is indirect (via Th17 recruitment). Moreover, it has been shown that inflammatory factors contribute to the initiation and progression of breast cancer. Chronic inflammation can initiate a series of molecular processes that result in the malignant transformation of differentiated cells, suppression of anti-tumor immunity, and the development and spread of tumors. The pro-inflammatory IL-17 is strongly related to breast cancer. It plays a role in growth, invasion, metastasis, and poor prognosis. Recent studies indicated a strong correlation between IL-17 and programmed death ligand 1 (PD-L1) in breast cancer. Studies have shown that the immune response, especially in triple-negative breast cancer in its early stage, is mediated by IL-17A. The IL-17A-mediated inflammatory response declines after surgery; however, IL-17A serum levels remain elevated compared with those in healthy controls, even after tumor resection. The IL-17A not only mediates the inflammatory response, but it also promotes breast cancer growth and progression [146,147]. Higher IL-17 levels are linked to the increased aggressiveness of invasive ductal carcinoma (IDC) of the breast. Metastatic primary tumor-infiltrating lymphocytes T produce increased levels of IL-17, while neutralizing IL-17 reduces tumor growth and blocks the migration of neutrophils and tumor cells to secondary sites. Pro-tumor neutrophils drive disease progression by producing CXCL1, MMP9, VEGF and TNFα, while their depletion inhibits tumor growth. IL-17A alters the gene expression profile and behavior of nonmetastatic tumor cells, promoting tumor growth in vivo and confirming its pro-tumor role. Moreover, high expression of IL-17 was associated with reduced disease-free survival and poorer prognosis in IDC patients [148].

IL-17, particularly IL-17A, plays a crucial role in breast cancer progression as it is a pro-inflammatory and tumor-promoting cytokine. Increased IL-17 levels are linked to more aggressive disease and poorer prognosis in IDC patients. Targeting IL-17 could be a promising therapeutic approach to limit tumor progression and improve patient outcomes (Figure 1, Figure 2 and Figure 3A) (Table 1).

## 19. Interleukin 18 (IL-18)

IL-18 is a member of the IL-1 family. It is a pleiotropic cytokine that induces interferon-γ [149]. It was first described in 1995. IL-18 has been described as an inducer of interferon-γ production in Kupffer cells, macrophages, and T and B cells. It is initially synthesized as an inactive 24 kDa precursor protein. It is then primarily acted on by caspase-1 and, to a lesser extent, proteinase-3 to produce the final mature, biologically active 18 kDa form. The regulation of IL-18R expression by TNF-α may play an important role in amplifying early inflammatory events, ultimately leading to IL-8 release in synovial fibroblast. Other sources of the cytokine may include adipocytes, dendritic cells, and osteoblastic matrix cells. Additionally, IL-18 is an important regulatory factor that inhibits osteoclastogenesis by inducing osteoprotegerin (OPG) expression. IL-18 also shows similarities to IL-1β in its ability to activate NF-kB, express FasL, and induce TNF-α, and regulates the immune response, making it potentially important in the pathogenesis of autoimmune diseases [150]. IL-18 levels were found to suppress an early stage of breast cancer bone metastasis in mice [151]. Liu et al. showed that IL-18 expressed by mesenchymal stem cells inhibits breast cancer cells’ proliferation in vitro [152]. IL-18 was, however, found to correlate positively with lymph node metastasis in breast cancer [153] (Figure 1 and Figure 2) (Table 1).

## 20. Interleukin 19 (IL-19)

IL-19 is produced by monocytes and macrophages. Interestingly, secretion is also increased by IL-4 and IL-13 [154,155]. IL-19 is a homolog of IL-10. IL-19 has a positive effect on the production of IL-16 and TNF, and, by influencing Th2 lymphocytes, it increases IL-4 production [154,155]. IL-19 is considered an important prognostic marker and potential therapeutic target. IL-19 is associated with breast cancer progression, including increased metastatic potential [156,157]. Additionally, it is associated with an unfavorable prognosis. It exerts autocrine effects, directly promoting cell proliferation and migration, and indirectly promotes tumor growth by stimulating fibronectin expression and creating a favorable microenvironment [157]. IL-19 also increases mitotic activity in breast cancer cells. The mechanisms of action of IL-19 in breast cancer have recently been investigated both in vitro and in vivo [156,157] (Figure 1, Figure 2 and Figure 3A) (Table 1).

## 21. Interleukin 20 (IL-20)

IL-20 is a protein encoded by the *IL20* gene in humans. IL-20 is part of the IL-10 cytokine family. IL-20 is produced by keratinocytes [158,159] IL-20 has a receptor, IL-20R. IL-20 plays a role in inflammation, the development of skin diseases, angiogenesis, and other pathological processes, including cancer [158,159,160,161]. IL-20 is an important factor influencing the progression of breast cancer, including the formation of metastases, especially to bones [162]. High expression of IL-20 and its receptors (IL20RA/IL20RB) in breast cancer tissues correlates with an advanced stage of cancer, increased ability to generate stem cells, and poor patient survival [163,164]. IL-20 promotes the proliferation and migration of breast cancer cells [163,164]. IL20RA signaling is highly expressed in breast cancer, promoting cancer stemness and apoptosis resistance, in part by associating with the transcription factor SOX2 [163]. Additionally, high expression occurs in breast cancer that has metastasized to the bone in murine models [164]. Moreover, research suggests that using an anti-IL-20 monoclonal antibody (e.g., 7E) can reduce tumor growth and inhibit the migration of breast cancer cells [161,165] (Figure 1, Figure 2 and Figure 3A) (Table 1).

## 22. Interleukin 21 (IL-21)

IL-21 is a cytokine belonging to the hematopoietins. IL-21 is produced by CD4+ T cells in response to antigenic stimulation. It binds to a receptor found on the surface of NK cells, B lymphocytes, activated T lymphocytes, and dendritic cells [166,167,168]. Interleukin-21/IL-21 belongs to the family of cytokines that bind to a complex receptor composed of a private receptor (IL21R) and a gamma chain common to several cytokine receptors [168]. The IL-21/IL-21R interaction activates JAK1 and JAK3 tyrosine kinases, and then activates STAT1 and STAT3 transcription factors [169,170]. IL-21 plays many important roles in the human body, mainly by enhancing the antigen-specific response of immune system cells. It stimulates the proliferation of previously activated B and T lymphocytes, and additionally inhibits B lymphocyte division. It does not affect NK cell proliferation, although it increases their cytotoxicity [168]. In addition, together with IL-15, it participates in the formation of NK cells in the bone marrow. Interestingly, it reduces IgE production. IL-21 also participates in the induction of anti-tumor response through the activation of CD8+ T lymphocytes and NK cells [171]. IL-21 plays a dual role in breast cancer. It acts as both a potential anticancer agent, stimulating NK and T lymphocytes to kill cancer cells, and, at times, promotes tumor growth by affecting B lymphocytes and the tumor microenvironment. Its effects vary depending on the breast cancer subtype and expression level [172]. Combining IL-21 with anti-HER2 antibodies (trastuzumab) shows promising results in overcoming resistance and increasing therapeutic efficacy, particularly in HER2-positive breast cancer in a mouse model [173] (Figure 1 and Figure 3B) (Table 1).

## 23. Interleukin 22 (IL-22)

IL-22 is a cytokine from IL-10 family of cytokines. IL-22 is produced by immune cells (Th17, Th22, and NKT lymphocytes). IL-22 binds to a receptor composed of two subunits (IL-22R1 and IL-10R2), activating the JAK-STAT signaling pathway. It does not act directly on immune cells but rather on structural (epithelial) cells, stimulating their proliferation, survival, and the production of antimicrobial peptides. It may be pro-inflammatory in autoimmune diseases or anti-inflammatory, supporting tissue homeostasis. It also protects against infections [174]. Elevated levels of IL-22 have been associated with the progression of breast cancer [175]. A group of Th cells producing IL-22 in breast and lung tumors is associated with poor patient prognosis. A constitutional and a T-specific knockout of IL-22 in mouse models of lung and breast cancer led to a reduction in metastasis without influencing the growth of primary tumor. Similarly, removing the IL-22 receptor from cancer cells resulted in a comparable decrease in metastasis as seen in mice lacking IL-22 [176] (Figure 1, Figure 2 and Figure 3B) (Table 1).

## 24. Interleukin 23 (IL-23)

IL-23 is a heterodimer consisting of two subunits: p19 (IL-23α/IL23A) and p40 (IL-12p40/IL12B) [177]. IL-23 binds to a heterodimeric receptor complex consisting of IL12RB1 and IL23R [177,178]. IL-23 activates JAK2/TYK2-STAT3 signaling, promoting Th17 cell survival and IL-17/IL-22 production from memory CD4^+^ T cells. IL-23 is essential for Th17 cell maintenance and IL-17A production, driving neutrophil infiltration and tumor progression [177,178,179]. Additionally, it participates in responses against bacteria, fungi, and extracellular parasites [177]. IL-23, together with IL-17, can act on the acute response to infection in peripheral tissues [180,181]. Additionally, it may be responsible for autoimmune inflammatory diseases and participates in tumorigenesis. In breast cancer, IL-23 promotes tumor growth and metastasis and suppresses the immune response by recruiting M2 macrophages and inhibiting T cell responses [182]. However, higher levels are often associated with larger tumors and advanced disease stages. Numerous studies indicate that the IL-23/IL-23R pathway is a potential target for new therapies. Post-chemotherapy IL-23 elevation in TNBC may correlate with improved anti-PD-1 responses, potentially reflecting restored T cell function. IL-23, an immunological cytokine, is significantly upregulated after chemotherapy in TNBC cells. Moreover, the combination of IL-23 and PD-1 mAb could synergistically inhibit the expression of phosphoinositide-3-kinase regulatory subunit 1 (PIK3R1). It is a regulatory subunit of PI3K that inhibits p110 activity and promotes AKT in TNBC-specific cytotoxic T cells (CTLs) [179,183]. Currently, we have access to two anti-IL-23 drugs: ustekinumab and risankizumab. Ustekinumab is a biologic drug, a human monoclonal antibody that specifically binds to the p40 subunit of IL-12 and IL-23 proteins. It is used in the treatment of inflammatory diseases, including plaque psoriasis, psoriatic arthritis, Crohn’s disease, and ulcerative colitis [184]. Risankizumab, a biologic drug, works by blocking IL-23 (anti-IL-23p19). It is used to treat inflammatory diseases such as plaque psoriasis, psoriatic arthritis, and Crohn’s disease. The satisfactory efficacy and tolerability of anti-IL-23 drugs in inflammatory diseases may provide a basis for studies of their efficacy in breast cancer treatment [185] (Figure 1 and Figure 3B) (Table 1).

## 25. Interleukin 24 (IL-24)

IL-24 (formerly melanoma differentiation-associated gene 7, *MDA-7/IL24*) was cloned in 1995–1996 and extensively studied since the early 2000s. Produced by immune cells (monocytes, T lymphocytes) and keratinocytes, it signals through the IL-20/IL-22 receptors, activating the JAK/STAT pathway. IL-24 is a recently identified tumor suppressor cytokine that specifically triggers apoptosis in various cancer types, including breast cancer [186]. The results demonstrate that IL-24 regulates apoptosis by influencing the phosphorylation of eukaryotic initiation factor 2 alpha (elF2α) under endoplasmic reticulum (ER) stress in cancer [187]. IL-24 triggers apoptosis in breast cancer cells by inducing G2/M phase cell cycle arrest and upregulating tumor suppressor proteins such as p53 and p27Kip1 [188]. Moreover, recombinant adenoviruses expressing IL-24 (e.g., Ad-mda7) have demonstrated selective killing of breast cancer cells in both in vitro and in vivo models, with minimal impact on normal cells [188,189]. IL-24 has been shown to inhibit cancer stem cell phenotypes and overcome resistance to conventional therapies [190] (Figure 1, Figure 2 and Figure 3B) (Table 1).

## 26. Interleukin 25 (IL-25)

IL-25, or more commonly known as interleukin-17E (IL-17E), is a distinct member of the IL17 cytokine family. It consists of at least six members that share a conserved cysteine knot structure but differ at the N-terminus [191]. IL-25 is produced by type 2 helper cells (Th2), mast cells, and other cells, such as epithelial cells, and stimulates them to produce other type 2 cytokines such as IL-4, IL-5, and IL-13 [192]. It also stimulates IL-8 production and can induce NF-κB activation [192]. The cytokine receptor IL17RB has two ligands, IL-25 and IL17B [193]. IL-25 plays a key role in the immune response [193,194]. This cytokine is associated with chronic inflammation of the gastrointestinal tract [192]. In addition, IL-25 was produced by tumor-infiltrating CD4+ T cells and macrophages. An IL-25-neutralizing antibody reduced lung metastasis but had no effect on primary tumor growth. Inhibition of IL-25 decreased T cells and macrophages in the primary tumor microenvironment, which may promote breast tumor invasion and metastasis to the lung. The study was conducted in a mouse model [195]. Moreover, IL-25 binds to IL-25R (IL-17RB) on breast cancer cells, inducing caspase-mediated apoptosis. Studies show that it specifically kills cancer cells without harming nonmalignant mammary epithelial cells [196] (Figure 1, Figure 2 and Figure 3B) (Table 1).

## 27. Interleukin 26 (IL-26)

IL-26 belongs to the IL-10 cytokine family. It has an amphipathic structure, participates in inflammatory signaling via a typical receptor pathway, and binds extracellular DNA to support cellular signaling and trigger intracellular inflammatory responses. IL-26’s involvement in cancer remains largely undefined. Nevertheless, certain studies have shown this cytokine to be one of the most significant inflammatory mediators involved in mammary engraftment and lung metastasis in triple-negative breast cancer (TNBC), which indicates that it can also serve as a therapeutic target in TNBC and other IL-26-dependent diseases [197]. Other in vitro studies have shown that IL-26 activates protein kinase B (AKT) and c-Jun N-terminal kinase (JNK) signaling pathways, which serve as alternative routes that enable TNBC cells to bypass the effects of EGFR tyrosine kinase inhibitors (EGFR-TKI’s). That phenomenon leads to tumor growth in TNBC patients; the restriction of IL-26 was proven to overcome it [198] (Figure 1, Figure 2 and Figure 3B) (Table 1).

## 28. Interleukin 27 (IL-27)

IL-27 is a part of IL-12 family and comprises two subunits: p28 (also known as IL-30) and Epstein–Barr virus-induced gene 3 (*EBI3*). That cytokine promotes Th1 differentiation, inhibits proliferation of Th2, and enhances cytotoxic T lymphocyte activity, while also helping to induce IFN-γ production [199,200]. Studies have indicated that serum levels of IL-27 are considerably elevated in breast cancer patients; it was stated that they are correlated with the clinical stage of cancer. Additionally, circulating IL-27 levels fell significantly following radical mastectomy. IL-27 acts as both a potential promoter of tumor growth, increasing PD-L1 expression in B cells and a tumor suppressor, increasing T cell activity and influencing tumor blood vessels. Data also shows that levels of IL-27 were especially increased in ER+ and PR+ (estrogen and progesterone receptor-positive) breast cancer [199]. High levels of IL-27 often correlate with poor survival and tamoxifen resistance [201] (Figure 1 and Figure 3B) (Table 1).

## 29. Interleukin 28 (IL-28)

IL-28, also referred to as IFN-λ (IL-28A as IFN-λ2 and IL-28B as IFN-λ3), is a protein encoded by genes located on human chromosome 19 [202]. Murine experiments using fibrosarcoma tumor models revealed that IL-28 released by tumor cells suppressed tumor growth and lung metastases [203]. Conversely, the release of IL-28 by Myeloid-Derived Suppressor Cells (MDSCs) affects STAT3 and promotes angiogenesis, tumor cell migration, and epithelial–mesenchymal transition (EMT), as shown in a study using a co-culture of canine mammary cancer cells with MDSCs [204]. IL-28 shows potential as an anticancer agent by activating immune cells such as natural killer cells and reprogramming Tumor-Associated Macrophages from M2 to M1 [205] (Figure 1, Figure 2 and Figure 3B) (Table 1).

## 30. Interleukin 29 (IL-29)

IL-29 is another cytokine belonging to the IFN-λ family and exhibits antiviral activity [206]. It was demonstrated that IL-29 promotes cell apoptosis and inhibits cell proliferation, thereby inhibiting cervical cancer cell growth [207]. Studies reveal that although IL-29 can express inhibiting effects in certain cancer types, such as lung or colorectal cancer, it also has pro-tumor effects in multiple myeloma [206]. In vitro studies showed that IL-29 plays a dual role in breast cancer, potentially inhibiting tumor development by inducing apoptosis and cell cycle arrest, but its effects can vary between cell types, sometimes promoting tumor progression [208] (Figure 1, Figure 2 and Figure 3B) (Table 1).

## 31. Interleukin 30 (IL-30)

IL-30 is a p28 component of interleukin 27 [209,210]. IL-30 belongs to the IL-12 cytokine family, which regulates both pro- and anti-inflammatory immune cell responses. IL-30 function intersects with that of IL-27, its precursor. It was shown that IL-30 plays an anti-inflammatory function analogous to that of IL-27. It has been discovered that IL-30 is derived from activated myeloid cells, which are its main cellular source. However, it may also be produced by neoplastic cells, particularly in aggressive tumors, which was observed in breast and prostate cancer. Experimental models and clinical samples suggest that IL-30, through interactions with myeloid cells, promotes the tumor microenvironment and accelerates tumor progression. Recent studies have found that IL-30 is an essential regulator of breast and prostate cancer metastasis. It shows that activated myeloid cells are not only the source of that chemokine, but also its target [210].

In the case of breast cancer, studies have shown that IL-30 levels are increased in HER2+ tumors and are linked to their recurrence and stage of disease. In an experiment using mice, it was shown that those injected with HER2+ cells and treated with IL-30 demonstrated enhanced proliferation and vascular spread of breast cancer cells. The conclusion was reached that it could be valuable in developing therapeutic monoclonal antibodies for cancer treatment [209]. IL-30 is usually not detected in normal mammary ducts, ductules, or histologically healthy breast tissue, but was observed in a few stromal leukocytes. A negative association was noted between IL-30 expression in stromal leukocytes and overall survival [211]. It has been demonstrated that IL-30 is often expressed by leukocytes infiltrating the tumor and lymph nodes in the case of TNBC. The absence of endogenous IL-30 inhibits the growth and progression of TNBC and extends the patient’s survival [212] (Figure 1, Figure 2 and Figure 3B) (Table 1).

## 32. Interleukin 31 (IL-31)

IL-31 is a protein encoded in humans by the *IL31* gene (locus 12q24.31) with a mass of 15.8 kDa and 141 amino acid residues [213]. IL-31 is a member of the gp130/IL-6 (glycoprotein 130) cytokine family, which is associated with its structural similarity to IL-6. IL-31 is a cytokine expressed by T cells. IL-31 transduces signals through a heterodimeric receptor consisting of the IL-31 receptor A (IL-31RA) and the oncostatin M receptor (OSMR) [213,214]. Subunits of this receptor are expressed on activated monocytes and unstimulated epithelial cells [213]. IL-31 is a cytokine with a four-helix bundle motif. It is produced by CD4+ T cells [213]. IL-31 is thought to play a role in the development of skin inflammation and may be associated with mastocytosis and the induction of pruritus in patients with mastocytosis [215,216]. The role of IL-31 in breast cancer slows tumor growth by promoting anti-tumor immunity through the stimulation of cytotoxic T lymphocytes and the inhibition of immunosuppressive cells [217]. The next study showed that the silencing of IL31RA suppresses the cancer stem cell-like properties, migration, and invasion of basal-like breast cancer (BLBC) cells in vitro as well as tumor growth and metastasis in vivo. Knockdown of IL31RA ameliorates IL-31-mediated pro-oncogenic functions. Studies show that high IL-31 expression correlates with improved patient survival [218] (Figure 1, Figure 2 and Figure 3B) (Table 1).

## 33. Interleukin 32 (IL-32)

IL-32 is a cytokine associated with various diseases as well as inflammatory conditions [219]. It has nine different isoforms [220]. An increase in IL-32 levels has been observed in numerous diseases, including systemic lupus erythematosus, HIV infection, and rheumatoid arthritis [220,221]. Together with interleukin-34, IL-32 plays an important role in promoting the progression of hepatocellular carcinoma [221]. Studies aimed at determining the impact of IL-32 on breast cancer cells have shown that it stimulates tumor progression, both in vitro and in vivo. The research demonstrated a link between IL-32 and the survival and proliferation of cancer cells, suggesting that IL-32 may become important in the treatment of breast cancer [222]. Anti-inflammatory properties of this cytokine have also been described. Its intracellular form is involved in defense against intracellular pathogens and in regulating cellular metabolism [219] (Figure 1, Figure 2 and Figure 3B) (Table 1).

## 34. Interleukin 33 (IL-33)

IL-33 is an immunomodulatory cytokine. It belongs to the IL-1 family [223]. The IL-1 family also includes IL-1β and IL-18. IL-33 consists of 160 amino acids with a molecular mass of 18.1 kDa. IL-33 is highly expressed in endothelial and epithelial cells. IL-33 acts as an extracellular cytokine and also translocates to the nucleus, where it inhibits gene expression [223,224]. IL-33 induces a type 2 T helper cells (Th2) response via the ST2 receptor. IL-33 was initially described as a nuclear repressor factor and later identified as an extracellular ligand for ST2 [225]. The ST2 receptor is encoded by the *IL1RL1* gene. IL-33/ST2 is involved in the development of asthma, rheumatoid arthritis, inflammatory bowel disease, Alzheimer’s disease, and carcinogenesis [225]. In breast cancer, IL-33 promotes tumor growth, metastasis, and therapy resistance by affecting the immune microenvironment. It increases the number of cancer stem cells and influences cell signaling. The expression of IL-33 was studied in tumor tissue and in serum from breast cancer patients [226]. Furthermore, elevated IL-33 levels have been shown to often signal a poorer prognosis [227] (Figure 1, Figure 2 and Figure 3B) (Table 1).

## 35. Interleukin 34 (IL-34)

IL-34 has three receptors: CSF-1R, syndecan-1, PTP-ζ. IL-34 primarily signals through CSF-1R, which it shares with M-CSF, and has also been reported to interact with PTPRZ1 (PTP-ζ) as an alternative receptor; syndecan-1 may function as a low-affinity binding partner, while TREM2 is not considered a direct IL-34 receptor (effects attributed to TREM2 are thought to occur indirectly in myeloid/microglial contexts involving DAP12 and CSF-1R signaling interplay). IL-34 possesses pro-inflammatory properties and plays an important role in the progression of inflammation and tumor development [228]. Studies have shown that its expression is increased in many diseases, including cancers, leading to resistance to therapies by creating an immunosuppressive tumor microenvironment and inhibiting angiogenesis [229]. In some pathological conditions, IL-34 expression is decreased. In vitro and in vivo studies conducted by Poudel et al. demonstrated that, through activation of the MEK/ERK and JNK/c-Jun pathways, IL-34 induces the transformation of epithelial cells, subsequently leading to breast cancer development [230]. High levels of IL-34 in TNBC (mouse model) are associated with poor outcomes because they create an immunosuppressive tumor environment, promote tumor cell survival, and cause resistance to chemotherapy [231] (Figure 1, Figure 2 and Figure 3B) (Table 1).

## 36. Interleukin 35 (IL-35)

IL-35 is a heterodimeric cytokine composed of two subunits: IL-12p35 (p35) and Epstein–Barr virus-induced protein (Ebi3) [232]. These genes encode distinct signal peptides; it has been suggested that p35 and Ebi3 are not secreted as the heterodimeric cytokine IL-35, but rather that these subunits act as independent anti-inflammatory cytokines; however, they mutually support each other’s secretion from cells [232,233]. Studies have shown that this interleukin can alter the mRNA profiles of small extracellular vesicles (sEVs), which in turn leads to activation of the Ras/Raf/ERK signaling pathway [234]. It follows that IL-35 indirectly affects endothelial function in breast cancer cells through the production of exosomes (IL-35-sEVs), resulting in increased cell proliferation and angiogenesis of human umbilical vein endothelial cells (HUVECs) [234,235]. In breast cancer, IL-35 promotes tumor growth and progression by suppressing anti-tumor immunity [236]. Higher levels of IL-35 in breast cancer patients are associated with advanced disease, larger tumors, and poorer prognosis [237] (Figure 1 and Figure 3B) (Table 1).

## 37. Interleukin 36 (IL-36)

IL-36 belongs to the IL-1 superfamily. Among the IL-36 cytokines, IL-36 alpha, IL-36 beta, and IL-36 gamma act as agonists, while IL-36Ra functions as an antagonist. Proteins of the IL-36 subfamily are important regulators of the innate immune system [238,239,240]. IL-36γ plays a key role in promoting a pro-inflammatory phenotype, which is particularly important for the anti-tumor immune response. Studies have shown that IL-36γ in the tumor microenvironment delays tumor progression by recruiting T cells and forming tertiary lymphoid organs (TLOs) [239,240]. IL-36 plays a dual role in breast cancer, acting as both tumor-promoting and tumor-suppressing factors. Studies show that IL-36γ can activate pathways such as MEK/ERK and JNK, stimulating tumor cell proliferation, while other studies suggest that it enhances anti-tumor immunity by activating T cells and natural killer cells [241,242] (Figure 1, Figure 2 and Figure 3B) (Table 1).

## 38. Interleukin 37 (IL-37)

Interleukin-37 (IL-37) is a member of the IL-1 family. Known as FIL-1ζ/IL-1H4/IL-1H/IL-1RP1. IL-37 can be induced by several Toll-like receptor (TLR) agonists and pro-inflammatory cytokines, such as IL-1β, tumor necrosis factor (TNF)-α, and interferon (IFN)-γ in peripheral blood mononuclear cells (PBMC) and dendritic cells (DC) [243]. Mature IL-37b can translocate to the nucleus via a caspase-1-dependent process. IL-37b can act as a cytokine with both intracellular and extracellular functionality. IL-37 has been implicated in autoimmune diseases at multiple levels [243,244]. IL-37, in breast cancer, inhibits tumor growth by enhancing anti-tumor immunity [245] (Figure 1, Figure 2 and Figure 3B) (Table 1).

## 39. Interleukin 38 (IL-38)

IL-38, also known as IL-1F10, is a member of the interleukin-1 (IL-1) family and also of the interleukin-36 (IL-36) subfamily. IL-38 has a similar three-dimensional structure to the IL-1 receptor antagonist (IL-1Ra) [246]. The IL-38 gene is located on chromosome 2q13. IL-38 binds to the IL-1 receptor type I. This cytokine plays an important role in inflammation and host defense, and is involved in the regulation of both innate and adaptive immunity [246,247,248]. Expression of IL1F10, encoding IL-38, correlated negatively with survival in breast cancer patients in the METABRIC22 dataset. Furthermore, there was a trend toward a stronger negative association of IL-38 with survival in triple-negative breast cancer (claudin-low, normal, and basal-like subtypes) [249]. In subsequent studies, treatment with antibodies against IL-38 has been shown to inhibit tumor growth in a syngeneic model of breast cancer. An effective response is associated with the development of immunological memory that inhibits subsequent tumor regrowth in the absence of additional therapy [250] (Figure 1, Figure 2 and Figure 3B) (Table 1).

## 40. Interleukin 39 (IL-39)

IL-39 is a member of the IL-12 family. It consists of the subunits IL-23p19 and Ebi3. IL-39 is produced by B lymphocytes. Additionally, IL-2p19 mRNA is produced by dendritic cells and macrophages [251]. IL-39 participates in inflammatory responses by binding to the IL-23R/gp130 receptor heterodimer and by acting on activator of transcription (STAT)1/STAT3. IL-39 has been shown to be associated with acute coronary syndrome [251,252]. Studies suggest that IL-39, together with microRNA-1246 (miR-1246) and HOX transcript antisense RNA (HOTAIR), can serve as an early diagnostic biomarker of breast cancer [253]. Laboratory studies have shown that IL-39 can directly inhibit the growth of breast cancer cells and promote their death [254] (Figure 1, Figure 2 and Figure 3B) (Table 1).

## 41. Interleukin 40 (IL-40)

IL-40, also called C17orf99, is involved in the function and formation of B cells and IgA. It is produced mainly in the bone marrow and fetal liver, and its expression can be induced in B lymphocytes upon their activation. IL-40 is not currently assigned to a known cytokine family. Research on IL-40 is still ongoing, and it is suggested that it may be important in certain diseases of the immune system [255,256]. We found no data in the current literature on the role of IL-41 in breast cancer (Figure 1) (Table 1).

## 42. Interleukin 41 (IL-41)

IL-41, also known as Metrnl/IL-41, is one of the most recently discovered interleukins. It is expressed by various cell types, including monocytes. It was characterized as an adipokine because it was highly expressed in adipocytes and adipose tissue [257,258]. It has also been implicated in adipose tissue function, insulin resistance, and metabolic and immunological diseases. Studies in mice have shown that IL-41 deficiency leads to decreased immunity. IL-41 may be a marker of anti-inflammatory response [257,258]. We found no data in the current literature on the role of IL-41 in breast cancer (Figure 1) (Table 1).

## 43. Clinical Trials

We searched the ClinicalTrials.gov database for registered trials using interleukin-based agents and different databases for the available results of these studies. Clinical trials of interleukins in breast cancer are mainly phase I and II and include metastatic breast cancer. Some studies include more than one organ in testing the efficacy of cytokine-based drugs (Table 2).

## 44. Functional Roles of Interleukins in the Breast Cancer Lifecycle

Interleukins in breast cancer can be functionally categorized into three groups: inducers of epithelial–mesenchymal transition (EMT) and invasion (e.g., IL-1, IL-6, IL-7, IL-28), regulators of cancer stem cells (e.g., IL-1, IL-6, IL-33), and mediators of the pre-metastatic niche (e.g., IL-11, IL-20, IL-30). The induction of EMT by interleukins occurs through several molecular pathways. One pathway involves STAT3 activation, where IL-6 upregulates EMT-inducing transcription factors such as Snail and Twist [67]. Another pathway is NF-κB signaling, activated by IL-1, which induces the expression of Snail and other transcription factors that drive EMT [23]. Additionally, the PI3K/Akt and Notch/TGF-β pathways, activated by IL-7, promote invasive properties [259]. Feedback loops also play a role; for example, IL-6 can activate a STAT3/miR-34a feedback loop, in which miR-34 repression is required for IL-6-induced EMT [67,68,260]. Cancer stem cells are regulated by inflammatory cytokines (IL-6, IL-1, and IL-33) and signaling pathways within the tumor microenvironment (TME) that promote their survival, self-renewal, and resistance to therapy. Several key signaling pathways are involved in this process. NF-κB is activated by IL-6, IL-1, and IL-8, creating a positive feedback loop to maintain stemness [23,261]. The second factor, STAT3 is activated by IL-6 to drive expression of stem genes [69,262,263]. STAT3, activated by IL-6, drives the expression of stem cell genes [69,262,263]. ST2-dependent signaling, particularly in response to IL-33, activates pathways that promote tumor growth [264,265]. NOTCH signaling, regulated by IL-6/Jagged1-Notch1, is essential for stem cell maintenance [266,267,268]. Interleukins also mediate the formation of the pre-metastatic niche (PMN), a supportive environment in distant organs that facilitates the survival and growth of circulating tumor cells prior to their arrival. Interleukins contribute to PMN formation by promoting immunosuppression, inflammation, and angiogenesis. PMN can be established through mechanisms such as myeloid cell recruitment, angiogenesis, immunosuppression, and extracellular matrix (ECM) remodeling. IL-1β and other factors promote the migration of CD11b+ myeloid cells, neutrophils, and macrophages to the pre-metastatic site. IL-6 and other cytokines induce vascular remodeling and increase permeability, facilitating cancer cell escape. Furthermore, interleukins promote a shift in the local immune environment toward an M2 macrophage and N2 neutrophil phenotype, which inhibits anti-tumor immune responses. Finally, interleukin signaling contributes to ECM remodeling by promoting the deposition of matrix components and the activation of enzymes that create a favorable environment for cancer cell attachment [269].

## 45. The Role of Interleukins in the Development of Breast Cancer Bone Metastases

Approximately 15% of breast cancer patients develop bone metastases. These metastases can result in hypercalcemia, bone fractures, spinal cord compression, pain, and impaired limb mobility. Pain arises from mechanical pressure exerted by the tumor mass and the release of inflammatory cytokines from tumor cells or the bone microenvironment, which disrupts bone homeostasis. Preclinical studies have identified several key interleukins (IL-1β, IL-2, IL-6, IL-8, IL-10, IL-11, IL-15, IL-17, IL-18, and IL-20) that promote breast cancer bone metastases. However, only IL-2 and IL-12 have been evaluated as therapeutic agents in clinical trials [254]. These interleukins exhibit anti-metastatic effects and have the potential to generate localized and systemic anti-tumor responses, although clinical trial results are not yet available. Breast cancer bone metastases are primarily osteolytic, but osteoblastic or mixed lesions may also occur. Tumor cells stimulate osteoclast activity and/or inhibit osteoblast differentiation, leading to increased bone resorption and/or decreased bone formation. Interleukins also modulate bone homeostasis. Elevated levels of IL-1β have been detected in breast cancer bone metastases in both in vitro and in vivo models. IL-1β is stimulated by osteoprotegerin (OPG) via the p38 and p42/22 mitogen-activated protein kinase (MAPK) signaling pathway, independent of breast cancer cell subtype, and contributes to invasion and promotion. Additionally, IL-1β regulates IL-11 and IL-8 production via TGF-β signaling pathways [270]. In vivo studies have shown that the IL-1R receptor antagonist (Anakinra) and, in vitro, the anti-IL-1β antibody (Kanakinumab) inhibit breast tumor growth and progression to bone metastases [271]. IL-6 is a key interleukin involved in the development of breast cancer and bone metastases. Elevated IL-6 levels have been observed in blood samples from patients with metastatic bone disease and in the serum of mice with bone metastases. High IL-6 levels also correlate with increased expression of Y-box binding protein 1 and multifunctional heat shock protein [272]. Subsequent studies have demonstrated higher IL-6 levels in the osteoporotic microenvironment. Several preclinical studies have elucidated mechanisms by which IL-6 influences the development of bone metastasis. For example, tumor cells expressing Jagged1 induce osteoblasts to express high levels of IL-6, whereas IL-6 production by osteoclasts is unaffected. Osteoblast-derived IL-6 inhibits osteoblast function and stimulates osteoclastogenesis, regardless of the presence of the receptor activator of nuclear factor kappa-Β (RANK)/RANK ligand (RANKL) pathway. IL-6 induces RANK expression in breast cancer cells, sensitizing tumors to RANKL and enhancing IL-6 release by tumor cells. RANKL and IL-6 mediate direct paracrine and autocrine signaling between osteoblast-lineage cells and tumor cells, promoting the growth of metastatic breast tumors in bone [273]. Disrupting this crosstalk by silencing IL-6 or RANK in breast cancer cells, or by treatment with antibodies against the IL-6 receptor or antagonist molecules such as TB-2-081, significantly reduces breast cancer growth in bone. This approach also diminishes the growth of breast cancer cells in hormone-refractory metastatic breast cancer, where anti-IL-6 receptor therapy blocks the IL-6hi/estrogen (ER)lo feedback loop [274,275]. Another mechanism involves signal transducer and Activator of Transcription-3 (pSTAT3). TB-2-081-induced IL-6 blockade increases pSTAT3 phosphorylation in breast cancer cells, thereby reducing osteolytic bone remodeling. Upregulation of Notch-3, Jagged-1, and carbonic anhydrase IX has also been correlated with breast cancer cell growth and bone invasion [254,276]. Metastatic breast cancer cells directly induce osteoblasts to express increased levels of IL-8, independent of the RANK/RANKL pathway particularly under osteoporotic conditions [276]. Osteoblasts release several growth factors, such as Transforming Growth Factor-β1 (TGF-β1), which activate activator protein 1 (AP-1) and nuclear factor kappa-light-chain-enhancer of activated B cells (NF-κB), initiating IL-8 release and promoting breast cancer cell migration and osteoclastogenesis. In vitro and in a mouse model of skeletal metastasis, both soluble Sema4D (semaphorin 4D) and its protein form, an immune semaphore expressed by T lymphocytes, eosinophils, dendritic cells, and B lymphocytes, produced by the breast cancer cell line, inhibit osteoblast differentiation. Additionally, Sema4D-mediated induction of IL-8 and LIX/CXCL5 (C-X-C motif chemokine 5), the murine homolog of IL-8, increases the number and activity of osteoclasts [254]. IL-11 is an osteolytic factor produced by breast cancer cells. In human breast cancer cells, IL-11 expression is regulated by runt-related transcription factor 2 (Runx2). IL-11 induction is regulated by TGF-β via signaling pathways such as SMAD (small mothers against decapentaplegic), p38 MAPK, and specific microRNAs (miRNAs) in metastatic breast cancer cells. Another mechanism of IL-11 induction involves the JAK1/STAT3 (Janus kinase/signal transducer and activator of transcription) pathway [277]. IL-11 induces osteoclastogenesis by activating the JAK1/STAT3 signaling pathway, which upregulates c-Myc expression. c-Myc is essential for osteoclastogenesis and acts independently of RANKL. Additionally, evidence indicates that IL-11 promotes RANKL-induced osteoclast differentiation [113,254].

## 46. Discussion

Interleukins are messengers between immune cells that stimulate cell growth, differentiation, and activation. They regulate inflammation, immune responses, and hematopoietic processes. They also participate in the development of malignant tumors, including breast cancer. In our review, we aimed to systematize and summarize all knowledge about all interleukins currently described in the literature regarding their impact on breast cancer development. We found no information on the influence of IL-14, IL-40, and IL-41 on breast cancer development, likely because these interleukins are still poorly understood. Interleukins may influence both the local development of breast cancer and be involved in metastatic disease. Knowledge about interleukins is constantly increasing, but despite numerous successes in in vitro and in vivo research, it has not yet been possible to introduce interleukins into breast cancer therapy. This may be due to several reasons.

Interleukins frequently act within complex molecular pathways, which may contribute to therapeutic failures. Blocking a single component of a signaling pathway may not achieve complete inhibition, as some pathways are activated by multiple interleukins or by independent factors. Interleukin actions involve activating or inhibiting immune system components, which can affect both malignant and healthy tissues, potentially resulting in significant treatment toxicity. For example, IL-2 was used in the treatment of kidney cancer and melanoma, but this approach was discontinued due to excessive toxicity [41]. Another limitation in the application of interleukins for breast cancer therapy is the variability of their effects depending on the molecular subtype of breast cancer. Most available data pertain to the effects of interleukins on triple-negative breast cancer (TNBC), possibly due to the aggressiveness of this subtype. The predominance of single-track studies may also hinder progress in understanding the role of interleukins in breast cancer development. Future studies should stratify by molecular subtype to enhance reliability. Numerous studies have demonstrated subtype-specific differences in interleukin effects. For instance, ER-negative cells secrete higher levels of IL-6 than ER-positive cells, as the estrogen receptor suppresses IL-6 expression by inhibiting NF-κB transactivation [63]. Increased IL-8 expression is observed in ER-negative and HER2-positive types [83]. IL13Ralpha2 is significantly elevated in basal-type breast cancers compared to primary luminal tumors [128]. IL-16 is considered a risk factor for HER2-positive breast cancer [139]. Data also indicate that IL-27 levels are particularly elevated in ER-positive and PR-positive breast cancer [199]. IL-30 levels are increased in HER2-positive tumors and are associated with disease recurrence and progression [209]. Additionally, IL-30 is frequently expressed by tumor- and lymph node-infiltrating leukocytes in TNBC [212].

Another limitation to the use of interleukins in breast cancer therapy is the presence of single-nucleotide polymorphisms (SNPs) in certain interleukins. SNPs in IL-10 and IL-12 resulted in differences in occurrence across breeds. A meta-analysis showed that the IL-10 rs1800896 and rs1800872 (AA vs. CC; A vs. C) polymorphisms significantly increased breast cancer risk in Asians. On the other hand, rs1800871 and rs1800872 (A vs. C) polymorphisms were associated with the risk of breast cancer in Caucasians [95]. Genetic variation in the IL-12 pathway has been associated with altered breast cancer risk in several populations, including Puerto Rican women [120].

Some interleukins share receptors or act synergistically with other interleukins, complicating therapeutic strategies that target a single interleukin. For example, the interleukin-4 receptor also binds to IL-13 [49]. IL-7, together with IL-15, stimulates breast cancer cell invasion [259]. Additionally, IL-10 inhibits the synthesis of other cytokines, including IL-1α, IL-1β, IL-6, IL-8, IL-12, and IL-18 [100]. IL-25, produced by Th2 helper cells, mast cells, and epithelial cells, stimulates the production of IL-4, IL-5, and IL-13, as well as IL-8, and may induce NF-κB activation [192].

Most data on the effects of interleukins comes from in vitro and in vivo studies. Despite many promising results from these studies, they have not yet translated into success in human trials. Clinical studies mainly focus on IL-1 and IL-2, with individual studies on IL-4/IL-13, IL-7, and IL-11. As we have already written, IL-2 was introduced with great hope for success in the treatment of kidney cancer and skin melanoma. However, this treatment failed because the toxicity of the treatment outweighed the benefits, leading to its withdrawal [39,41]. Therefore, when considering therapy with interleukins in breast cancer, possible toxicities should be taken into account and minimized as much as possible.

Currently, therapies targeting IL-1 and IL-23 appear most promising, as these approaches have demonstrated success in other diseases. Studies have shown that Anakinra or Canakinumab can prevent bone metastases in breast cancer patients, although in some cases this was accompanied by primary tumor growth. This phenomenon likely reflects the complex mechanisms by which interleukins influence breast cancer development. Further evidence indicates that targeting IL-1 signaling with MLX01, an inhibitor of IL1β secretion, reduces both primary tumor growth and bone metastases [35]. These findings underscore the complexity of interleukin action within molecular pathway cascades. Two anti-IL-23 drugs, ustekinumab and risankizumab, are currently used to treat inflammatory diseases and may serve as a foundation for research into their efficacy in breast cancer treatment [184,185].

Interleukins may also serve as diagnostic, predictive, or prognostic markers in breast cancer. For instance, IL-3 expression is associated with poor treatment outcomes in breast cancer patients [48]. Serum IL-12 has been investigated as a potential diagnostic or prognostic marker, with lower levels associated with more advanced disease or a poorer prognosis [121]. Overexpression of IL13Ralpha2 is associated with poorer survival in patients with nonmetastatic breast cancer compared to those with low expression [126]. Increased IL-16 expression correlates with tumor growth inhibition and improved prognosis [139]. Elevated IL-17 levels are associated with increased aggressiveness in invasive ductal carcinoma (IDC) of the breast [148]. High expression of IL-20 and its receptors (IL20RA/IL20RB) in breast cancer tissues correlates with advanced cancer stage, enhanced stem cell generation, and poor patient survival [161]. Overexpression of IL-27 in serum correlates with tumor stage, whereas IL-31 is associated with improved patient survival [201,218]. Elevated levels of IL-33 and IL-35 are frequently linked to advanced disease, larger tumors, and poorer prognosis [227,237]. Additionally, the combination of IL-39, miR-1246, and HOTAIR may serve as an early diagnostic biomarker for breast cancer [254].

Certain interleukins can positively or negatively influence current and potential breast cancer therapies. IL-7 enhances the therapeutic effects of CAR-T cells, particularly in triple-negative breast cancer [81]. IL-17 is strongly correlated with programmed death ligand 1 (PD-L1) expression in breast cancer [146,147]. Combining IL-21 with anti-HER2 antibodies (trastuzumab) has shown promise in overcoming resistance and increasing therapeutic efficacy, especially in HER2-positive breast cancer [173]. Increased IL-23 levels following chemotherapy may correlate with improved anti-PD-1 responses and restored T cell function. The combination of IL-23 and an anti-PD-1 monoclonal antibody in TNBC can synergistically inhibit phosphoinositide 3-kinase regulatory subunit 1 (PIK3R1) expression [179,183]. IL-24 inhibits cancer stem cell phenotypes and overcomes resistance to conventional therapies [190]. IL-27 plays dual roles: as a tumor growth promoter by increasing PD-L1 expression in B cells and as a tumor suppressor by enhancing T cell activity and influencing tumor vasculature. High IL-27 levels are often associated with poor survival and tamoxifen resistance [199]. Elevated IL-34 levels in TNBC are linked to poor prognosis due to the creation of an immunosuppressive tumor environment, promotion of tumor cell survival, and induction of chemotherapy resistance [231]. Similar findings have been reported for IL-10 in ovarian cancer, where patients refractory to anti-PD-1/anti-PD-L1 monotherapy exhibit associations between IL-10, PD-1, and PD-L1 signaling pathways. Blocking both PD-1 and IL-10 reduces tumor burden and enhances the immune response, thereby improving prognosis in ovarian cancer. These numerous examples of interleukin interactions with PD-1 or PD-L1 suggest potential directions for future research to enhance the effectiveness of interleukin therapy in breast cancer [100,109].

Some interleukins exhibit differential effects on distant metastases compared to primary tumors, an important consideration for study design. For example, reducing IL13Ralpha2 expression in metastatic breast cancer cells slightly delayed primary tumor growth but significantly inhibited lung metastasis [128]. Similarly, administration of IL-22 in mouse models of lung and breast cancer reduced metastases without affecting primary tumor growth [176].

Further preclinical studies are essential to advance understanding of the cellular and molecular relationships and signaling pathways of individual interleukins. The evidence presented highlights the significant potential of interleukins in breast cancer treatment, although the aforementioned limitations must be carefully considered.

## 47. Conclusions

Since 1863, we have known that the inflammatory process is involved in the pathogenesis of malignant tumors, thanks to the research of Virchow R. Numerous studies have also confirmed this in breast cancer. One of the key factors of the immune system responsible for the inflammatory process in malignant tumors is interleukins. All clinical trials conducted to data using interleukins as a therapeutic or diagnostic targets have failed. These studies were completed in phase I or II. Currently, there are several open clinical trials that give hope for the use of interleukins as a therapeutic target in breast cancer, most of which concern TNBC. However, the latest studies are focused on multiple therapeutic targets simultaneously to enhance interleukin effects, which may be the key to success. Many studies are behind us, but there is still a lot of work ahead of us to determine the actual role of interleukins in the development of breast cancer and to use it correctly as a therapeutic or diagnostic target.

## Figures and Tables

**Table 1:** Source cells and the main function of interleukins implicated in breast cancer.

Interleukin	Receptor	Source Cell	Target Cell	Main Function in Breast Cancer
IL-1	IL-1RA IL-1RI IL-1RII	monocytes, tissue macrophages, dendritic cells, B lymphocytes, natural killer (NK) cells, epithelial cells	Mesenchymal stem cells, Myeloid cells, Endothelial cells, fibroblasts	promotes tumor growth, angiogenesis, metastasis, immunosuppression, development of a hostile tumor microenvironment
IL-2	IL-2R IL-2Rα (CD25) IL-2Rβ (CD122) IL-2Rγc (CD132)	activated T cells, particularly CD4+ and CD8+ T cells, activated dendritic cells, natural killer cells	Natural Killer (NK) Cells, CD8+ T cells, regulatory T cells (Tregs), Tumor-Infiltrating Lymphocytes	promotes tumor growth, stimulates the immune response, suppresses the immune response, promotes apoptosis
IL-3	IL-3Rα (CD123)	T lymphocytes	Tumor-derived Endothelial Cells (TECs), Stromal Cells, Tumor-Infiltrating Immune Cells	angiogenesis, metastasis, stimulates the immune response, promotes apoptosis
IL-4	IL-4R IL-4Rα IL-4Rγc	Th2 cells, mast cells, eosinophils, and basophils	Macrophages, Myeloid Suppressor Cells	promotes tumor growth, angiogenesis, metastasis, stimulates the immune response, promotes apoptosis
IL-5	IL-5RA IL-5Rβ	Th2 CD4+ lymphocytes and mast cells	eosinophils, neutrophils, CD4+ T cells (Th2), B Cells	promotes tumor growth, metastasis, stimulates the immune response
IL-6	IL-6R (CD126)	macrophages and monocytes	Immune Cells in the tumor microenvironment (TME), Stromal fibroblast, endothelial cells	promotes tumor growth, metastasis, stimulates the immune response, immunosuppression therapeutic resistance
IL-7	IL-7R IL-7Rα IL-7Rγc	stromal cells in the bone marrow and thymus	T Lymphocytes, Cancer-Associated Fibroblasts (CAFs), Engineered CAR-T cells	promotes tumor growth, stimulates the immune response,
IL-8	CXCR1 i CXCR2	monocytes and neutrophils, as well as epithelial, fibroblast, and endothelial cells	Endothelial Cells, neutrophils, macrophages, Cancer-Associated Adipocytes (CAAs)	promotes tumor growth, angiogenesis, metastasis, promotes apoptosis
IL-9	IL-9R	mast cells, NKT cells, and type 2 innate lymphoid cells (ILC2s)	CD8 + T cells, Mast Cells, Immuno Microenvironment	promotes tumor growth, metastasis, inhibits of metastasis
IL-10	IL10RA IL10RB	T cells, macrophages, monocytes, and dendritic cells	Tumor-Associated Macrophages (TAMs), Dendritic Cells (DCs), T cells (CD8+ and Treg Cells)	promotes tumor growth, immunosuppression, angiogenesis, stimulates the immune response, inhibits angiogenesis
IL-11	IL-11RA	bone marrow cells, stromal cells, and immune cells	Osteoclast Progenitors (Bone), Immune Cells (Tumor Niche), Osteoblasts (Bone)	promotes tumor growth, angiogenesis, metastasis
IL-12	IL-12Rβ1 IL-12Rβ2	antigen-presenting cells (APCs) like dendritic cells (DCs), monocytes, and macrophages	Natural Killer (NK) Cells, CD8+ T cells, CD4+ Th1 Cells, Antigen-Presenting Cells (APCs), Tumor-Associated Macrophages (TAMs)	improves tumor microenvironment, inhibits angiogenesis, promotes apoptosis
IL-13	IL-13R IL13RA1	activated T helper type 2 (Th2) cells	Tumor-Associated Macrophages (TAMs), Immune Cells Microenvironment	promotes tumor growth, metastasis, stimulates the immune response
IL-14	IL-14R	There are no studies	There are no studies	There are no studies
IL-15	IL-15R IL-15Rα IL-15Rβ IL-15Rγc	dendritic cells, follicular dendritic cells	Natural Killer (NK) Cells, CD8+ T cells, Memory T cells	improves the tumor microenvironment, promotes tumor growth, metastasis
IL-16	CD-4	T cells (both CD4+ and CD8+), eosinophils, mast cells, dendritic cells, monocytes, fibroblasts, epithelial cells.	CD8+ T cells, Tumor-Associated Macrophages (TAMs), CD4+ T cells, Mast Cells	Promotes apoptosis, stimulates the immune response, metastasis
IL-17	IL-17RA IL-17RB IL-17RC IL-17RD IL-17RE	Th17 cells, γδ T cells, natural killer cells, and neutrophils	Tumor-Associated Macrophages (TAMs), Tumor Associated Neutrophils (TANs), Cancer-Associated Fibroblasts (CAFs)	promotes tumor growth, immunosuppressions, angiogenesis, chemoresistances, metastasis
IL-18	IL-18R: IL-18Rα (IL-1R7) IL-18Rβ (IL-18RAP or CDw218b)	macrophages, dendritic cells, monocytes, keratinocytes, mesenchymal cells.	Tumor-Associated Macrophages (TAMs), Immune Stromal Cells, Natural Killer (NK) Cells	promotes tumor growth, promotes apoptosis, metastasis
IL-19	IL20RA IL20RB	resident glial cells, particularly astrocytes	Tumor-Associated Macrophages (TAMs), T cells	promotes tumor growth, metastasis
IL-20	IL-20RA IL-20RB	myeloid cells such as monocytes, granulocytes, and dendritic cells, keratinocytes, fibroblasts	Endothelial Cells, Bone Cells (Osteoclasts), Tumor-Associated Macrophages (TAMs), Myeloid-Derived Suppressor Cells (MDSCs)	promotes tumor growth, metastasis, immunosuppressions, chemoresistances
IL-21	IL-R21 IL-21Rα IL-21Rγc	CD4+ helper T cells, particularly follicular helper T cells (Tfh cells)	NK Cells, CD8+ T cells, B Cells, CD4+ T cells	promotes tumor growth, inhibits tumor growth
IL-22	IL-22R IL-22R1 IL-10R2	T helper 1 (Th1) cells, T cell subsets like Th17 and Th22 cells	Stromal Cells	promotes tumor growth, metastasis
IL-23	IL-23R	dendritic cells, macrophages	Macrophages, neutrophils, regulatory T cells (Tregs), Cancer Stem Cells (BCSCs), Tumor Cells	promotes tumor growth, immunosuppressions, metastasis
IL-24	IL-20R1 IL-20R2 IL-22R1 IL-20R2	immune and non-immune cells, including myeloid cells, lymphoid cells, and epithelial cells	Tumor Cells, Cancer Stem Cells (BCSCs)	promotes apoptosis, inhibits angiogenesis, inhibits metastasis
IL-25	IL-25R IL17RA IL-17RB	immune and non-immune cells	Tumor Cells, Stromal Fibroblasts	promotes tumor growth, angiogenesis, promotes apoptosis, inhibits angiogenesis, inhibits metastasis
IL-26	IL-20R1 IL-10R2	T helper 17 (Th17) cells, natural killer (NK) cells, macrophages, and fibroblast-like	monocytes, macrophages, neutrophiles, CD4+ T cells, TNBC cells	promotes tumor growth, metastasis
IL-27	IL-27Rα (also known as WSX-1 or TCCR) and gp130	antigen-presenting cells (APCs) such as dendritic cells, macrophages, and monocytes, Myeloid-Derived Suppressor Cells, CD4+ and CD8+ T cells, osteoclasts, and activated B cells.	Cancer Cells, Cancer- Associated Fibroblast	promotes tumor growth, angiogenesis, stimulates the immune response.
IL-28	IL-28Rα	dendritic cells (DCs), and regulatory T cells, and it is also produced by activated monocytes and macrophages.	Natural Killer	reduces tumor growth, promotes apoptosis, promotes metastatis and inhibits metastasis, radiosensitization, immune modulation
IL-29	IL-29R	maturing dendritic cells, macrophages, and monocytes	Breast Cancer Cells, Cancer-Associated Fibroblasts (CAFs)	promotes tumor growth, metastasis, stimulates the immune response
IL-30	unknown	monocytes, macrophages, and dendritic cells	Stem Cells, Tumor Cells, Immune Cells, Stromal Cells	promotes tumor growth, metastasis
IL-31	IL-31RA OSMRβ	T cells, particularly Th2 helper cells, mast cells, macrophages, dendritic cells.	Macrophages, Myeloid-Derived, Suppressor Cells (MDSCs), CD8+ T cells, CD4+ T cells, Breast Cancer Cells	reduces tumor growth, promotes tumor growth
IL-32	unknown	natural killer (NK) cells, T cells, monocytes, and epithelial cells.	Breast Cancer Cells, macrophages, immune Cells,	promotes tumor growth, invasiveness, metastatis
IL-33	ST2 (also known as IL1RL1)	astrocytes, psoriatic keratinocytes, airway epithelial cells	Breast Cancer Cells, Myeloid-Derived Suppressor Cells, regulatory T cells, Tumor-Associated Macrophages, Fibroblast, Endothelial Cells, Innate Lymphoid Cells	promotes tumor growth, metastasis
IL-34	CSF-1R, syndecan-1, PTP-ζ and TREM2	monocytes, macrophages, microglia, neurons, epithelial cells, endothelial cells, fibroblasts, and hepatocytes	Tumor-Associated Macrophages, Breast Cancer Cells, Myeloid-Derived Suppressor Cells, T cells, NK cells	promotes tumor growth, inhibits tumor growth, stimulates the immune response.
IL-35	IL-12Rβ2/gp130	regulatory T cells (Tregs), but also by other immune cells like regulatory B cells (Bregs) and tolerogenic dendritic cells (tolDCs).	Tumor-Infiltrating Lymphocytes, Natural Killer Cells, Endothelial Cells, Macrophages	promotes tumor growth, angiogenesis, immunosuppression.
IL-36	IL-36R (IL1RAP and IL1RL2)	keratinocytes, monocytes, dendritic cells.	CD8+ T cells, Natural Killer Cells, Tumor- Associated Myeloid Cells, Breast Cancer Cells, Tumor-Associated Macrophages	promotes tumor growth, angiogenesis, inhibits metastasis, immunosuppression, stimulates the immune response.
IL-37	IL-18Rα and IL-1R8 (SIGIRR)	macrophages, dendritic cells, epithelial cells, certain types of T cells	CD4+ T cells, CD8+ T cells, Breast Cancer Cells, Tumor-Associated Macrophages, Mast Cells, Dendritic Cells	inhibits tumor growth, inhibits angiogenesis, inhibits metastasis, stimulates the immune response, angiogenesis, promotes tumor growth.
IL-38	IL-36R, IL-1R1, IL-1RAPL1	keratinocytes in the skin, specifically within the epidermis. It is also produced by various other cell types including B cells, fibroblasts, and immune cells like macrophages and dendritic cells	CD8+ T cells, Breast Cancer Cells, Macrophages, Mast Cells	promotes tumor growth, suppresses anti-tumor immunity.
IL-39	IL-23 R/gp130	activated B cells, macrophages	Breast Cancer Cells, Myeloid Cells,	inhibits tumor growth, promotes apoptosis, stimulates the immune response.
IL-40	unknown	activated neutrophils and B cells.	unknown	unknown
IL-41	unknown	entheseal stromal cells at the enthesis, bone marrow macrophages	unknown	unknown

**Table 2:** Clinical trials on ILs and breast cancer.

Type of Interleukin	Phase	Type of Breast Cancer	Trial Arms	Trial Number and Status
IL-7	II	Metastatic breast cancer	Interleukin 7-CYT107 (an immunotherapy by IL-7 on CD4 lymphopenia)	NCT01368107 Completed
IL-2	Observational	Metastatic breast cancer patients present with pleural effusion	IL-2	NCT01256801 Completed
IL-1 Alpha	I	Breast cancer: metastatic or locally advanced (Stage III/IV)	Interleukin 1-Alpha With Ifosfamide	NCT00001270 Completed
IL-7	Observational	invasive breast cancer, without distant metastasis	biomarker	NCT05300412 Completed
IL-2	I/II	IIIB or metastatic breast cancer.	combination of low dose interleukin-2 (IL-2) sargramostim (GM-CSF), and multiple doses of activated T cells (ATC) following peripheral blood stem cell transplantation	NCT00002780 Unknown status
IL-4/IL-13	Not Applicable	stage I-II breast cancer related lymphedema	QBX258, a combination of two fully human monoclonal antibodies that neutralize the biologic activity of interleukin 4 and interleukin 13	NCT02494206 Completed
IL-11	II	Breast cancer stage III or IV	recombinant interleukin-11 + filgrastim	NCT00004157 Completed
IL-2	IB/II	breast cancer amenable to anthracycline therapy.	F16IL2 in combination with doxorubicin	NCT01131364 Terminated
IL-2	IB/II	breast cancer amenable to taxane therapy.	F16IL2 in combination with paclitaxel	NCT01134250 Completed
IL-1	I	Metastatic breast cancer	Anakinra	NCT01802970 Completed
IL-2	I		RO6874281	NCT02627274 Completed
IL-1	II	Early TNBC	Anakinra (Kineret^®^)	NCT06710197 Recruiting
IL-2	I	Relapsed/Refractory Advanced or Metastatic NY-ESO-1 Overexpression Positive Triple-Negative Breast Cancer	Aldesleukin	NCT05989828 Recruiting
IL-7	I/II	Triple-Negative Breast Cancer	Efineptakin alfa (NT-17)	NCT04332653 Active, not recruiting
IL-7	I/II	Advanced and Metastatic Breast Cancer	MDK-703	NCT05716295 Terminated
IL-2	II	Triple-Negative Breast Cancer	Aldesleukim	NCT03449108 Active, not recruiting

## Data Availability

No new data were created or analyzed in this study. Data sharing is not applicable to this article.
